# Machine Learning in FTIR Spectrum for the Identification of Antibiotic Resistance: A Demonstration with Different Species of Microorganisms

**DOI:** 10.3390/antibiotics13090821

**Published:** 2024-08-30

**Authors:** Claudia Patricia Barrera Patiño, Jennifer Machado Soares, Kate Cristina Blanco, Vanderlei Salvador Bagnato

**Affiliations:** 1 São Carlos Institute of Physics, University of São Paulo, Avenida Trabalhador São-Carlense No. 400, Parque Arnold Schimidt, São Carlos CEP 13566-590, SP, Brazil; jennifer.soares@usp.br (J.M.S.); kateblanco@ifsc.usp.br (K.C.B.); 2 Biomedical Engineering, Texas A&M University, 400 Bizzell St., College Station, TX 77843, USA

**Keywords:** antibiotic-resistant bacteria, machine learning algorithms, *Streptococcus pyogenes*, *Streptococcus mutans*, *Escherichia coli*, *Klebsiella pneumoniae*

## Abstract

Recent studies introduced the importance of using machine learning algorithms in research focused on the identification of antibiotic resistance. In this study, we highlight the importance of building solid machine learning foundations to differentiate antimicrobial resistance among microorganisms. Using advanced machine learning algorithms, we established a methodology capable of analyzing the FTIR structural profile of the samples of *Streptococcus pyogenes* and *Streptococcus mutans* (Gram-positive), as well as *Escherichia coli* and *Klebsiella pneumoniae* (Gram-negative), demonstrating cross-sectional applicability in this focus on different microorganisms. The analysis focuses on specific biomolecules—Carbohydrates, Fatty Acids, and Proteins—in FTIR spectra, providing a multidimensional database that transcends microbial variability. The results highlight the ability of the method to consistently identify resistance patterns, regardless of the Gram classification of the bacteria and the species involved, reinforcing the premise that the structural characteristics identified are universal among the microorganisms tested. By validating this approach in four distinct species, our study proves the versatility and precision of the methodology used, in addition to bringing support to the development of an innovative protocol for the rapid and safe identification of antimicrobial resistance. This advance is crucial for optimizing treatment strategies and avoiding the spread of resistance. This emphasizes the relevance of specialized machine learning bases in effectively differentiating between resistance profiles in Gram-negative and Gram-positive bacteria to be implemented in the identification of antibiotic resistance. The obtained result has a high potential to be applied to clinical procedures.

## 1. Introduction

With the increase in antibiotic resistance cases around the world, the World Health Organization (WHO) is calling for joint efforts to combat antibiotic multi-resistance (AMR). In order to treat this emergence, it is necessary to include expertise from different fields, like our time’s science, data science, clinical studies, and public health among others; then, it is necessary to think about the different ways and methodological strategies to detect, identify, and develop a safe treatment to antibiotic resistance effectively [1,2,3,4]. Currently, the AMR can be solved using two branches: (i) the development of new methods to detect antibiotic resistance, and (ii) the development of new antibiotics to combat infections and diseases [5,6,7,8,9,10,11,12,13,14,15,16,17,18,19].

Due to the importance of this topic in global health, all the efforts in this research field are valid. Here, we are introducing our study about the identification of antibiotic resistance in Gram-positive and Gram-negative microorganisms. Here, we are introducing the results of our research actively exploring the broader applications of our approach [20]. At this point, we introduce the analyses of the Fourier Transform Infrared Spectroscopy (FTIR) structural profile spectra of the samples of *Streptococcus pyogenes* and *Streptococcus mutans* (Gram-positive), as well as *Escherichia coli* and *Klebsiella pneumoniae* (Gram-negative), with machine learning algorithms, on the database of *Staphylococcus aureus* [20]. 

The last can have significant implications for the new method that can bring accuracy to the identification of antimicrobial resistance in different microorganisms in quick and safe ways. This implies one consistent contribution to a new identification tool that can provide support in the management and treatment of bacterial infections. The current advances in data science and data analyses in machine learning have generated an innovative and promising research field [17,21,22,23]. This can be applied to the strategy of examining AMR and could provide a useful tool to bring support and treatment in the rapidest time for the patient, which can be highly significant in maintaining their health.

At this juncture, we developed the validation of our proposed methodology to identify antibiotic resistance through the means of the implementation of machine learning algorithms in the analyses of FTIR spectra from bacterium samples with/without susceptibility to antibiotics [20,24,25,26]. Our first bacteria analysis focused on both antibiotic-sensitive and resistant bacteria within the species *Staphylococcus aureus* [20] and on the identification of antibiotic resistance.

The phenomenon of bacterial antibiotic resistance development is intricate and multifaceted, exhibiting significant variance between the Gram-positive and Gram-negative groups, attributable to their inherent structural and physiological disparities. In Gram-positive bacteria, the dense peptidoglycan layer within the cell wall serves as a principal target for antibiotics that disrupt cell wall synthesis, such as penicillin. Resistance mechanisms can emerge through the enzymatic alteration of antibiotic targets within the cell wall, notably via the production of β-lactamases, enzymes that catalyze the breakdown of β-lactam antibiotics [27]. 

Conversely, Gram-negative bacteria are characterized by a thinner peptidoglycan layer, enveloped by an outer membrane comprising lipopolysaccharides (LPSs) and porins. The resistance mechanisms in Gram-negatives frequently entail alterations to the outer membrane’s permeability, including modifications or the reduction in porins, coupled with the activation of efflux pumps, which proficiently expel antibiotics from the cellular interior [28]. 

In a previous study [20] has been developed a methodology in which in the first stage, the system conforming by four specified species groups: Control, amoxicillin-induced (AMO), gentamicin-induced (GEN), and erythromycin-induced (ERY) was analyzed with success in three window intervals: Carbohydrates, Fatty Acids, and Proteins. Then, in the second stage, five hidden samples were identified and classified with (or not) resistance to antibiotic-induced correctly [20,24,25,26]. In all of them, the sample preparation and FTIR acquisition spectra follow the protocol explained in [24,25,26,29], the above by the statistical accuracy obtained to evaluate and test the supervised and unsupervised machine learning algorithms implemented in the prediction of antibiotic detection [20].

In this approach, distinct statistical processes can establish that the identification, detection, and prediction were performed successfully due to a significant portion of using the sample preparation methodology created in prior work [24,25,30,31]. To develop the correctness and the accurateness, calculations were implemented in the robust method, which is amplified in the section FTIR spectra database analysis process overview; some computational resources are also implemented to develop the analyses of samples due to the complexity of the system. 

In this study, the identification, detection, and prediction of susceptibility to antibiotic resistance to four microorganisms were analyzed. It was performed with the use of machine learning algorithms analyzing the samples in the interval windows of Carbohydrate (900–1200 cm^−1^), Fatty Acids (2800–3100 cm^−1^), and Protein (1500–1800 cm^−1^) in FTIR spectra.

The promising results obtained in the previous study [1] bring the methodology implemented here to develop the study for four different microorganisms. Machine learning algorithms were implemented here for the identification of antibiotic resistance, and the results obtained validated the procedure employed before [1]. In this study was obtained antibiotic identification with high accuracy, sensitivity, and susceptibility to four different species of microorganisms on a database of *S. aureus*. It lets us develop a demonstration of our methodology in the function of the analyses of FTIR spectra that work properly and safely independently of the microorganisms studied.

## 2. Results

In this study, we proved the methodology proposed in the previous study [20]. It was performed with the goal of the implementation of supervised and non-supervised machine learning algorithms. With these tools, it is recognized and characterized by biochemical profiles in the FTIR spectrum. This study was developed for the identification of resistance in Gram-positive and Gram-negative bacterium evaluated on *S. aureus* FTIR spectrum database. 

The data cleaning process was obtained using numerical tools in our codes developed in MATLAB (R2021b) [32] and it was applied to the database of FTIR spectra for all the samples studied. Data analyses and machine learning algorithms have been developed and built in the R Project for Statistical Computing (4.2.3) [33,34,35,36,37,38,39,40,41].

The data analysis methodology implemented here for *S. pyogenes*, *S. mutans*, *E. coli*, and *K. pneumoniae* study is joined with the implementation of machine learning algorithms. It is due to developing classification, clustering [2,3,4,5,6,7], principal component analysis (PCA) [1,8,9,10,11,12,13], and confusion matrix [1,14,15,16,17,18,19] on a database of *S. aureus* applied to a dataset of the four microorganisms introduced now. Calculations and results let us determine the class (antibiotic susceptibility/or not) for the samples analyzed with confidence values of probability and accuracy. 

The results of analyzing the FTIR spectrum of the four microorganisms studied here have been obtained in the investigation into the interval windows of Carbohydrates, Fatty Acids, and Proteins. Currently, the machine learning algorithms recognize the genuine features of each microorganism sample. The features are recognized from the specific categories of the species *S. aureus* [20] in which the database was trained.

The microorganisms presented in this study have been introduced with the aim of developing the identification of antibiotic resistance with machine learning algorithms on the *S. aureus* database. It was carried out for four microorganisms independently, in each FTIR interval window, to the species groups: Control, AMO, GEN, and ERY. The microorganisms were prepared with/without antibiotic susceptibility. Following the methodology developed previously [20], it was implemented in the study of twenty FTIR spectra for each microorganism: *S. pyogenes*, *S. mutans*, *E. coli*, and *K. pneumoniae.* The spectra acquisition maintained the same protocols shown in previous studies [1,20,21,22,23]. The statistical process implemented for the identification of antibiotic resistance from FTIR spectra is described in more detail in the methods section.

The compendium of the results obtained from the implementation of machine learning algorithms in the study is shown in Table 1. As a validation step for the machine learning results, Minimum Inhibitory Concentration (MIC) experiments were performed for the antibiotics amoxicillin, erythromycin, and gentamicin, and the representative results of identification to susceptibility to antibiotics in the microorganisms studied are shown in the Carbohydrate, Fatty Acid, and Protein intervals. 

### 2.1. Gram-Positives Species: S. pyogenes and S. mutans

**Figure 1:**
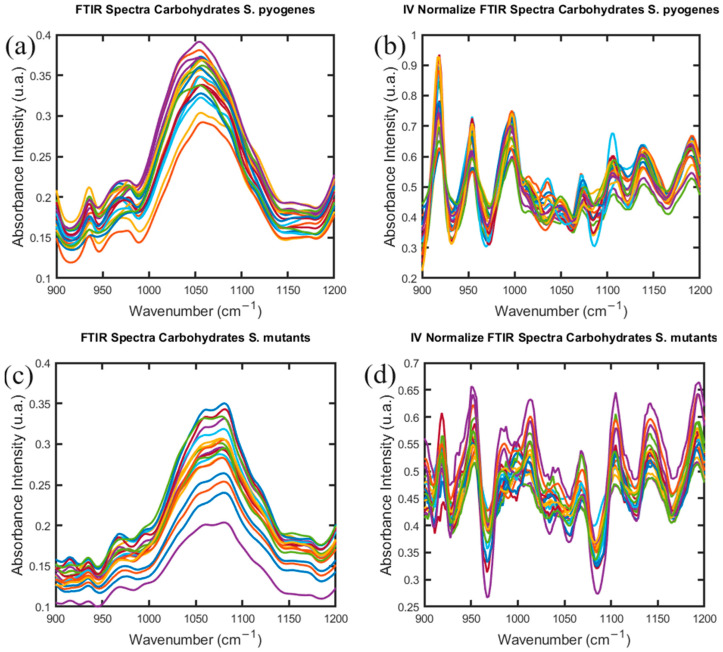
FTIR spectra in Carbohydrate interval windows of *S. pyogenes* (**a**,**b**) and *S. mutans* (**c**,**d**), with the normalized absorbance obtained to this interval region for each bacteria species.

The FTIR spectrum of *S. pyogenes* and *S. mutans* in the Carbohydrate interval windows are shown in Figure 1. Both the spectral profiles are not equivalent in shape. The total FTIR spectra for microorganisms are shown in Figure S1 in Supplementary Materials. Figure 1 reveals that the intensity and shape are different in various peaks and valleys. The FTIR spectrum of *S. mutans* and *S. mutans* in the Fatty Acid and Protein interval windows showed the same behavior (Figures S2 and S3 in Supplementary Materials).

Differences in the FTIR spectrum between the strains in terms of absorbance intensity have been detected. That is significant in the spectral derivative process (Figure S1 in Supplementary Materials). The result of this procedure has great interest due to its use in interpreting the spectra data to design and develop the analysis by machine learning algorithm. Here, it is conducted especially for this task in each interval window of our study.

Principal component analysis (PCA) was implemented in the analyses of forty FTIR spectra of the microorganisms *S. pyogenes* and *S. mutans*. The calculation was carried out in the Carbohydrate, Fatty Acid, and Protein window intervals. For both microorganisms, we obtained specific features and specific statistical behavior for the species: Control, AMO, ERY, and GEN that were used in the machine learning algorithm to develop the analyses.

**Figure 2:**
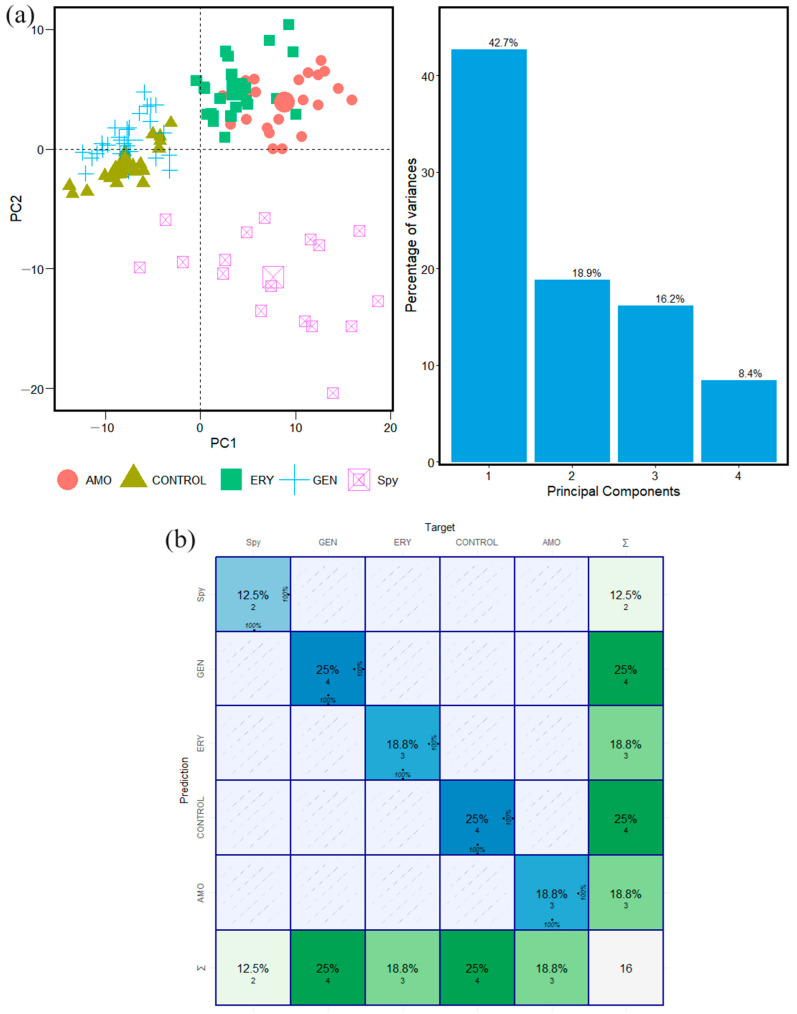
Classification steps and machine learning analyses for twenty FTIR spectra of *S. pyogenes* bacteria samples in Carbohydrate window interval. (**a**) PCA calculation and statistical variance results. (**b**) Confusion matrix calculation results.

**Figure 3:**
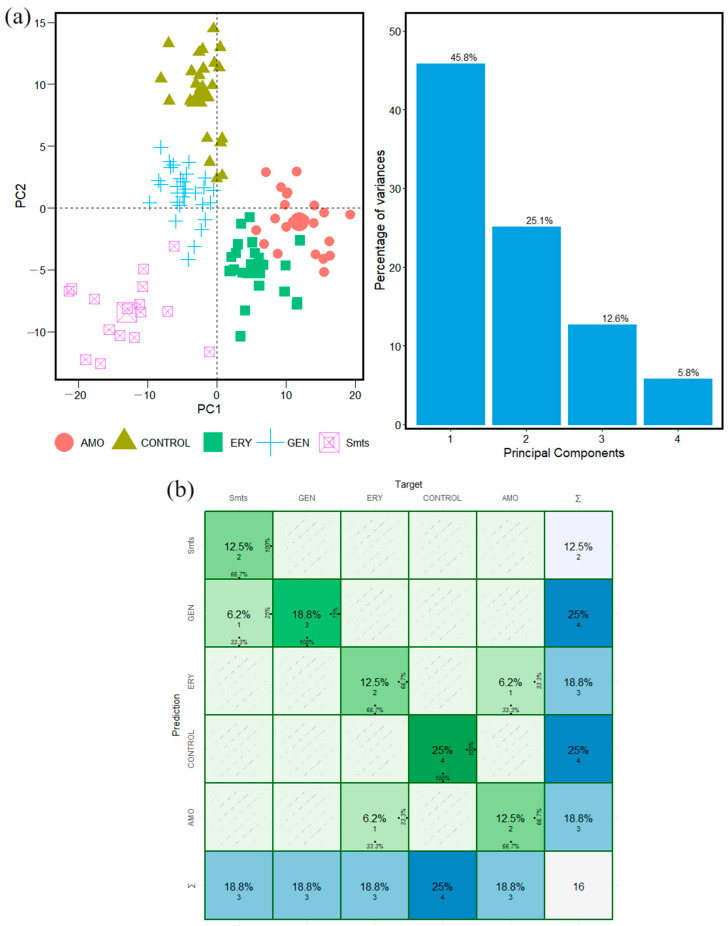
Classification steps and machine learning analyses for twenty FTIR spectra of *S. mutans* bacteria samples in Carbohydrate window interval. (**a**) PCA calculation and statistical variance results. (**b**) Confusion matrix calculation results.

The results obtained by applying PCA to the FTIR spectra of the microorganisms *S. pyogenes* and *S. mutans* in the Carbohydrate window interval are shown in Figure 2 and Figure 3, respectively. The results obtained vary between 60% and 90%, which means that the data are available and organized into the first and second principal components in all the cases with these values of variance (Figure 2 and Figure 3). Figures S5a, S6a, S8a and S9a in Supplementary Materials show PCA results in Fatty Acid and Protein intervals for the microorganisms *S. pyogenes* and *S. mutans*, respectively.

The spatial distribution and clustering arrays for control species and antibiotics in the Carbohydrate interval windows of *S. pyogenes* and *S. mutans*, in Figure 2 and Figure 3, show that the species have been well localized and are specifically differentiated from each other. It is one indicator that the analyses were performed in the proper way. The same behavior is observed in the Fatty Acid and Protein intervals. Each one has a different spatial distribution and statistically specific behavior of features associated with the microorganism studied (Figures S5a, S6a, S8a and S9a in Supplementary Materials). 

In addition, in the data analyses, studies with dendrograms were developed too for the FTIR spectra of the microorganisms *S. pyogenes* and *S. mutans* in the window intervals associated with Carbohydrate, Fatty Acids, and Protein (Figures S4 and S7, Supplementary Materials). The hierarchical results from the dendrogram are interpreted here using branches and nodes. Then, for each microorganism, each node indicates the similarity between the FTIR spectra that are joined to the branches. 

Also, the height of the nodes is linked to the difference between the groups, then higher nodes indicate greater dissimilarity. The dendrogram in the Carbohydrate, Fatty Acid, and Protein groups of the microorganisms *S. pyogenes* and *S. mutans* indicates cluster analysis results based on specific spectral signatures. It implies that within the studied microorganisms, there are significant variations in the Carbohydrate, Fatty Acid, and Protein composition or structure of the bacterial cells. 

The results from the calculation performed on the dataset of the microorganisms *S. pyogenes* and *S. mutans* with a confusion matrix are shown in Figure 2b, Figure 3b, Figures S5b, S6b, S8b and S9b for Carbohydrate, Fatty Acids, and Protein, respectively. This confirms the accuracy of the methodologic classification implemented in this study. The rows indicate the actual species, whereas the columns represent the expected species. The major diagonal indicates how many times each type was successfully identified, allowing for an examination of the prediction accuracy using data from a previous PCA in the FTIR window intervals studied here. 

To introduce the way in which the confusion matrix reports the calculation results, it is possible to say that the results of the calculation and identification of antibiotic resistance from FTIR absorption spectra are developed in the Carbohydrate, Fatty Acid, and Protein intervals. The calculations have been performed with forty FTIR spectra of *S. pyogenes* and *S. mutans* microorganisms. In addition, the process has been performed with Control, AMO, ERY, and GEN species to identify antibiotic resistance susceptibility existence/(or not). 

The last species has originated on a database built from a previous study developed with *S. aureus* bacteria [20] following the protocol developed by Soares et al. [24,25,26]. These calculations are developed with 80% of the dataset in the training group and 20% of the dataset conforming to the test group. Completed descriptions of the confusion matrix calculation for the species are available in the previous study [20]. 

The confusion matrix calculation results in the identification of antibiotic resistance are shown in a graphical representation of Carbohydrates, Fatty Acids, and Proteins in Figure 2b, Figures S4b and S5b, respectively. In addition, the data analysis results from the confusion matrix calculation are found in Table 1. The prediction result table is built on a base of results with high accuracy values. For example, in the Carbohydrate group, the accuracy value was one (Figure 2b). For Fatty Acids and Proteins, the accuracy values are between 0.812 and 0.954. 

In addition, the confusion matrix calculations are based on high values obtained from statistical parameters like sensitivity and specificity calculated independently for each one of the groups Control, AMO, ERY, GEN, and microorganism database. All the calculations have been developed in the window interval groups of the FTIR absorption spectra studied here in an independent way, taking a database set corresponding to each sample and each studied group, respectively, as was developed in previous research [20,24].

The statistical results identify specific components or patterns that differentiate microorganisms and species. This methodologic can uncover important aspects of the microorganisms *S. pyogenes* and *S. mutans* biology. And, they could be associated with metabolic adaptations or resistance mechanisms, but more studies are necessary to verify it.

### 2.2. Gram-Negative Species: E. coli and K. pneumoniae

**Figure 4:**
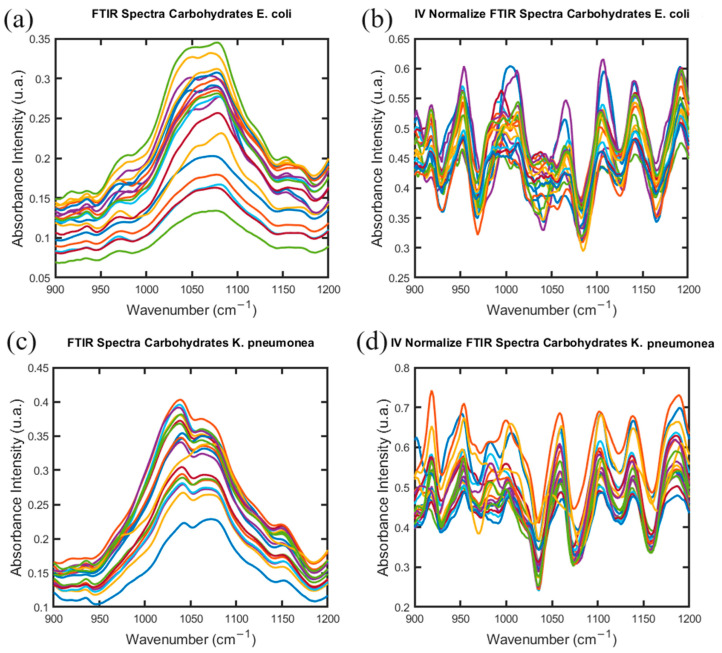
FTIR spectra in Carbohydrate interval windows for *E. coli* (**a**,**b**) and *K. pneumoniae* (**c**,**d**), with the normalized absorbance obtained in this interval region for each bacteria species.

The study of the microorganisms *E. coli* and *K. pneumoniae* to identify antibiotic resistance was developed with the same methodology that was described in the previous section. Also, forty FTIR spectra were acquired for each microorganism (Figure S10 in Supplementary Materials). The calculation was performed in the Carbohydrate, Fatty Acid, and Protein window intervals from the FTIR spectra in an independent way (Figure 4, Figures S11 and S12 in Supplementary Materials).

Based on the attributes of the vibrational modes of the Carbohydrate, Fatty Acid, and Protein biomolecules of *E. coli* and *K. pneumoniae* were built the dendrograms for each microorganism. The results of the hierarchical classification are shown in Figures S13 and S16 in Supplementary Materials for each microorganism studied in this section. 

**Figure 5:**
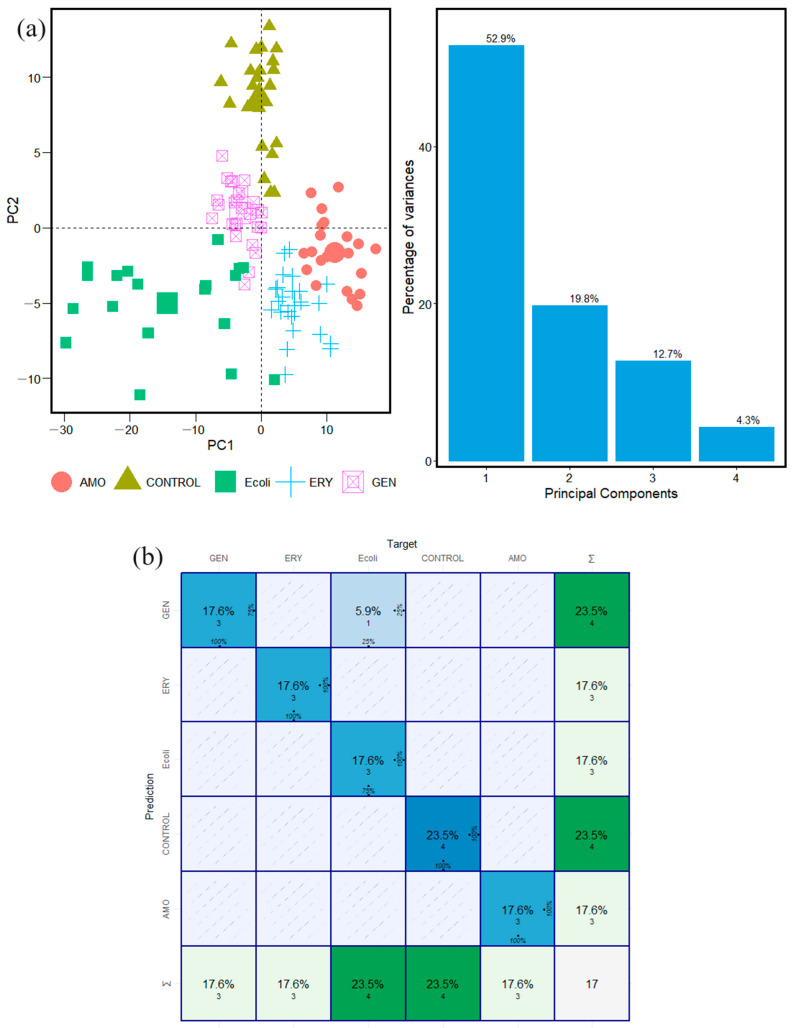
Classification steps and machine learning analyses for twenty FTIR spectra of *E. coli* bacteria samples in Carbohydrate window interval. (**a**) PCA calculation and statistical variance results. (**b**) Confusion matrix calculation results.

**Figure 6:**
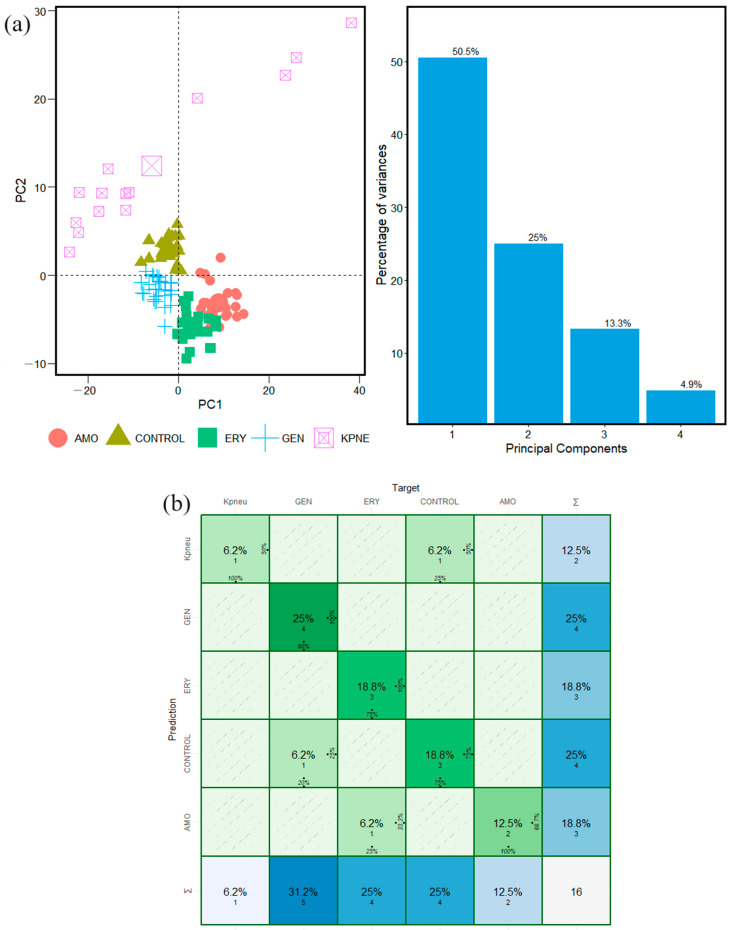
Classification steps and machine learning analyses for twenty FTIR spectra of *K. pneumoniae* bacteria samples in Carbohydrate window interval. (**a**) PCA calculation and statistical variance results. (**b**) Confusion matrix calculation results.

Along with that, with the microorganisms *S. pyogenes* and *S. mutans*, it was possible to develop the identification of the specific features in the FTIR spectra associated with the microorganisms. It was conducted in each window interval in an independent way. From Figure 5a and Figure 6a that show the PCA results, it is evident that the subpopulations are forming far away from the database of the species Control, AMO, GEN, and ERY built in a previous study of *S. aureus*. 

The last behavior was observed previously in Figure 2a and Figure 3a, but here in Figure 5a and Figure 6a, the distance between the clusters is more evident, especially the difference in spatial distribution of the samples, and the Euclidean distance between the center of mass of the cluster distribution of the microorganisms and species is increased. 

Also, there are differences in their characteristics, resulting in dispersion across different quadrants in the PCA. This behavior is observed also in the PCA results in the Fatty Acid and Protein window intervals of the FTIR spectra of the microorganisms *E. coli* and *K. pneumoniae* (Figures S14a, S15a, S17a and S18a in Supplementary Materials). The statistical results identify specific features that are associated with patterns that differentiate the microorganisms and species and identify their similarities to the groups in the database built in the previous study [20]. 

The graphical results of the confusion matrix calculations in the Carbohydrate, Fatty Acid, and Protein window intervals are shown in Figure 5b, Figure 6b, Figures S14b, S15b, S17b and S18b in Supplementary Materials. Thus, the confusion matrix identified similarities between the species Carbohydrates and Control (species without antimicrobial resistance). For Fatty Acids and Proteins, the similarity is found in erythromycin-induced resistance species.

The microorganisms studied here maintained the fingerprints of the vibrational modes of the biomolecules, resulting in an FTIR spectrum with similarities between them. The microorganism samples displayed well-defined categories in the data analyses developed with machine learning algorithms. The last is according to the statistical results from the dendrogram and PCA results. The data analysis reports variance values of at least 80% in the study developed in Carbohydrate, Fatty Acid, and Protein interval windows. 

In order to give a detailed description of the results and interpretation of them, here, we will take the microorganism *E. coli* to do the description. About the spatial distribution of the samples in the PCA, *E. coli* is mostly distributed in the third quadrant close to the gentamicin-induced resistance group in the Carbohydrate interval. It was verified and highlighted by the confusion matrix calculation results. In the Fatty Acid interval, the sample is distributed in the first, third, and fourth quadrants. But it is not close to specific antibiotic/(or not) species. The analysis from the confusion matrix reveals correspondence with the erythromycin-induced resistance group. 

Likewise, for the microorganism *E. coli*, the PCA in the Protein interval showed a spatial distribution in the first and third quadrants, also approaching different groups, though the confusion matrix refines the similarity with the amoxicillin-induced resistance group. The last calculation results were verified with the results from a microbiological study developed for the research involved in this study. It was performed with each microorganism to probe the results of all the calculations. All the results are reported in Table 1. 

## 3. Discussion

The FTIR data spectra were classified with machine learning algorithms because the datasets were built each by one spectrum in an accurate way. Due to the data acquired exhibiting null values of variance between them, all were used in the training, testing, and evaluating processes in the machine learning algorithms. 

The learning process involves estimating the parameters of probability distributions based on the dataset from the FTIR spectrum from each species group. The advantages of using FTIR spectroscopy are the safe and efficient way of sample signal collection [42]. FTIR spectra analysis can be used to distinguish between microorganisms [43] and strains [44,45], and to identify antimicrobial resistance [20]; the last one is the focus of this study. The identification of the structural profile of bacteria using FTIR was based on the analysis of the characteristic spectral peaks, which reflect the specific molecular vibrations of the different functional groups present in the molecules. These peaks provided valuable information about the chemical composition of the bacteria, including Carbohydrate, Fatty Acid, and Protein chemical groups. 

Regarding Proteins, the peaks corresponding to the Amide II band were the result of N–H deformation and C–N stretching vibrations and are related to the presence of Proteins and peptides. Fatty acids are represented by the C=O stretching vibrations of carbonyl groups present in Fatty Acids and are indicative of the presence of lipids and cell membranes. The Carbohydrate band is dominated by C–O stretching vibrations, C–O–H deformation vibrations, and C–C stretching vibrations [29,46]. These spectral signatures in this range are indicative of the presence of structural polysaccharides such as peptidoglycans in the bacterial cell wall and can also reveal information about glycoproteins and lipopolysaccharides in Gram-negative bacteria.

It is important to mention here that an accurate identification of antibiotic resistance by means of the analyses of FTIR spectra can become a big challenge. FTIR spectra obtained for the different microorganisms studied here are quite similar in shape, so then accurate tools are necessary to develop the analyses and identify small differences between them. 

Then, it is necessary to implement statistical procedures in the machine learning algorithms in an accurate way to detect possible differences between spectrums in a big volume of data. Also, it is required to develop this task with high precision. In addition, the methodology to advance in the identification of antimicrobial resistance includes the analyses of the features in the chemical representative groups of Carbohydrates, Fatty Acids, and Proteins in the FTIR spectra of each microorganism. 

The chemical intervals of Carbohydrate, Fatty Acid, and Protein groups play a crucial role in the results of the identification of antibiotic resistance. This is due to bacterial adaptability mechanisms in these intervals’ windows, which is a fundamental method that contributes to resistance, affecting the effectiveness of antibiotic treatments [47]. The adaptability creates variations that alter the bacterial physiology and morphology [47]. 

In addition, it also influences the interaction of these microorganisms with antimicrobial agents. For example, changes in the Fatty Acid composition of the membrane can modify cellular permeability, limiting the entry of antibiotics [48]. Resistant bacteria can alter the Fatty Acid chain to create a more effective barrier against the penetration of antimicrobial substances [5]. 

In Gram-positive bacteria, modifications in cell wall Carbohydrates, especially in peptidoglycan, can influence the binding of antibiotics that act to inhibit cell wall synthesis. The modification of the wall Carbohydrates may impede the action of such antibiotics [14]. Structural changes in target Proteins, such as penicillin-binding Proteins, can decrease the affinity of antibiotics, rendering them ineffective, commonly observed in β-lactam resistance [27].

In a previous study [20], we used supervised/unsupervised machine learning algorithms to train the database with the labeled FTIR spectra of *S. aureus* bacteria. It was performed with the purpose of developing the classification and identification of antibiotic resistance in five unknown samples. We obtained useful results with the implemented machine learning algorithms used to distinguish and identify antibiotic resistance strains based on the analyses of their unique FTIR spectral profiles. 

The analysis of the FTIR spectra data of Gram-positive and Gram-negative was performed with supervised learning methods [49]. The species data group in addition to Control, AMO, GEN, and ERY were tested and evaluated with statistical analysis procedures. It was performed to determine the accuracy of the classification process. The verification of the results obtained of each microorganism studied here was carried out too. Furthermore, each sample was evaluated at each window interval independently.

In the same way, the datasets of all the species were tested. The process was performed until obtaining high accuracy in the statistical parameters. This was performed so the algorithm learns, in an optimal way, to classify between the specific features of each microorganism because the biomolecule fingerprints themselves may be in different proportions and constitutions in each microorganism studied here. 

The classification of data from FTIR spectra is conducted using unsupervised machine learning algorithms. These are implemented here for hierarchical cluster analysis [1,2,3,4,5,8,24,25,26,27,28,29]. We applied it due to its specific characteristic to identify natural patterns, subgroups, or new classifications within complex bacterial datasets [50]. To assist in the identification of distinct patterns or biochemical signatures between samples is applied the classification tree [1,20,23,30,31]. It develops the differentiation between strains or identifies specific markers related to phenotypic characteristics, such as pathogenicity or antibiotic resistance [22,32,33,34,35,36,37,38,39,40]. This type of analysis is particularly valuable for accurate diagnosis in this case considering monitoring outbreaks and developing targeted therapies [50].

With the use of machine learning algorithms was carried out the recognition process of patterns in the FTIR spectra from each microorganism studied here. It proved the effectiveness of the machine learning algorithms implemented here in the identification of the susceptibility to antibiotics in specific bacterial strains. Although different bacterial species have distinct spectrum patterns, this study shows that a machine learning model designed for one species can successfully generalize to others, even across different phylogenetic levels (Gram-positive or Gram-negative).

The results obtained in this study showed that machine learning algorithms bring useful methodology to establish a protocol for the detection and identification of antibiotic resistance. It is performed in a safe, accurate, and not expensive way, which simply is a tool to establish the correct treatment for each patient individually in a quick simple way to gain time to benefit health. The use and implementation of these methods will allow the selection of optimized actions to treat antibiotic resistance in an individual way. It is likely to be effective, minimizing unnecessary antibiotic use and reducing the risk of developing new resistance [24,25,28]. Furthermore, it would help with the rapid diagnosis accelerating the identification of the susceptibility to antibiotics.

## 4. Materials and Methods

### 4.1. Samples Preparation and FTIR Spectra Acquisition

The susceptibility to antibiotic samples was prepared and the FTIR spectrum samples were acquired following the protocol based on Soares et al. [24,26]. The total analyses for the four samples, *Streptococcus pyogenes*, *Streptococcus mutans*, *Escherichia coli*, and *Klebsiella pneumoniae*, were carried out in three windows: Carbohydrates, Fatty Acids, and Proteins in FTIR spectra.

### 4.2. Microorganisms

The *S. pyogenes*, *S. mutans*, *E. coli*, and *K. pneumoniae* bacteria were reactivated from frozen stocks in cryotubes containing 20% glycerol. The samples were seeded on brain heart infusion (BHI) agar medium using the colony depletion technique and kept at 37 °C for 48 h.

### 4.3. Fourier Transformation Infrared Spectroscopy

Colonies from the plated samples were evenly distributed over the crystal surface. The FTIR absorption spectra of the bacteria were acquired in the window intervals: Carbohydrates, Fatty Acids, and Proteins [20,32,46]. In sum, one hundred FTIR absorption spectra formed by the samples of *Streptococcus pyogenes*, *Streptococcus mutans*, *Escherichia coli*, and *Klebsiella pneumoniae* bacteria were acquired in the FTIR equipment by Attenuated Total Reflection (ATR) on the Agilent Cary 630 FTIR Spectrometer^®^ instrument (Billerica, MA, USA) in the wavelength range of (650–4000) cm^−1^. All of them were acquired following the protocol developed by Soares et al. in [20,24,25,26].

### 4.4. FTIR Spectra Database Analysis Process Overview

The initial process involved the implementation of the statistical tools of the mean and variance of data from the FTIR spectra for the four bacteria *S. pyogenes*, *S. mutans* and *E. coli*, and *K. pneumoniae* bacteria. The dataset implemented in this study was developed from the FTIR spectra of *S. aureus* bacteria [20]. This dataset includes spectra from various conditions and treatment methods, focusing on capturing the diverse biochemical signatures associated with antibiotic resistance.

The last was built with the purpose of classifying, evaluating, and predicting antibiotic resistance in bacteria species for different classes of antibiotics, with accuracy from implemented statistics and machine learning algorithms. The statistical behavior in the dendrogram hierarchical results in clustering (Figures S4, S7, S13 and S16 in Supplementary Materials) using methods such as tree-like structures, k-means, k-medoids, hierarchical clustering, SSE (the sum of the squared distances of each point from its closest centroid), and calculate silhouette coefficient [20,49,51,52,53,54,55,56]. All of them were used to determine the optimal number of clusters in hierarchical clustering and classification/regression trees.

These statistical results are employed in the spatial distribution interpretation of the species in the PCAs (Figure 2, Figure 3, Figure 5 and Figure 6). The cluster conformation and spatial distribution are associated with the fingerprints in each data group of microorganisms analyzed and its connection with chemistry and biological characteristics.

Additionally, machine learning algorithms including supervised/unsupervised learning were applied for classification and prediction. Supervised/unsupervised learning algorithms learn from labeled/unlabeled data, respectively, to discover patterns and predict outcomes. Depending on the dataset and the work performed with that dataset was used Mean Squared Error (MSE), Mean Absolute Error (MAE), R-squared (R^2^), Silhouette Score, the Sum of squared distances, variance, and Adjusted Rand Index (ARI).

Also were implemented regression, classification, and clustering algorithms. The data analyses started with data cleaning and pre-processing. After that was implemented clustering, Random Forest, principal component analysis (PCA), principal component regression (PCR), probabilistic PCA (PPCA), Reinforcement Learning, and deep learning algorithms [16,19,20,30,31,33,37,49,52,57,58,59,60,61,62,63,64,65,66,67,68,69,70,71,72,73,74,75,76,77,78,79,80]. In addition, a confusion matrix was applied for classification and prediction data, and its performance was evaluated using metrics like accuracy, precision, recall, sensitivity, F1 score, loss function, and mean average precision (MAP) [31,34,55,69,78,81,82,83,84,85,86,87,88]. 

The statistics process developed in our study with machine learning algorithms, supervised and unsupervised, have been implanted in the evaluation of the performance of classifiers and statistical processes in all the steps developed in the algorithms. Working in this way, it is obtained that the classification and prediction process of machine learning has been developed with high accuracy, as shown in the previous work [20]. The efficiency of this method is reflected in the accuracy of the results obtained from our constructed database joined with our machine learning algorithms that came to the results about antibiotic resistance analyses and prediction from diverse samples and strains, with the final process through confusion matrix analysis.

With this statistically rigorous process applied to entire data analysis procedures by machine learning algorithm implemented here was developed the prediction process of the susceptibility and identification of antibiotic resistance to four microorganisms (*S. pyogenes*, *S. mutans*, *E. coli*, and *K. pneumoniae*) and previously to five hidden bacteria samples [20]. These uses for our constructed data bank provide high accuracy of our data and methods implemented in this study about antibiotic resistance susceptibility identification in bacteria. The ‘hidden samples’ referred to five previously unclassified bacterial strains that were used to validate the robustness of our classification model.

### 4.5. Methodology Developed to Find/Determine the Effect of the Antibiotics

The bacterium preparation and findings determine the effect of the antibiotics where the cultured microorganism samples follow the process implemented by Soares et al. [24,25,26]. Here, briefly, we comment that the resistance was induced by amoxicillin, erythromycin, and gentamicin by the cultivation in Mueller–Hinton medium with an antibiotic concentration of ¼ MIC for 72 h total, at 37 °C, 150 rpm to *Staphylococcus aureus* strain (ATCC 25923); for more information, please see Soares et al. [24,25,26] study results.

Bacterial susceptibility was previously assessed by determining the Minimum Inhibitory Concentration (MIC) for the antibiotic amoxicillin, erythromycin, and gentamicin. Various concentrations of these antimicrobials were added to a 96-well plate containing Mueller–Hinton medium. The bacteria were inoculated and incubated for 24 h at 37 °C to determine the lowest concentration that inhibited bacterial metabolic activity. The obtained values were then compared against the EUCAST (European Committee on Antimicrobial Susceptibility Testing) breakpoints.

In the current study, the database built with *S. aureus* FTIR spectra has been evaluated with the FTIR samples of Gram-positive and Gram-negative bacteria species. The identification of antibiotic susceptibility associated with a specific bacteria sample was developed in our first study with *S. aureus* [20]. Then, the database (conforming with nine hundred FTIR spectra) was verified with an excellent result in the calculation, identification, and prediction of antibiotic resistance to five hidden samples of bacteria. The goal of this study is to obtain the identification of antibiotic resistance with machine learning algorithms for these four bacteria in an accurate way.

Our results bring a safe alternative to the identification of antibiotic resistance in microorganisms (Figure 1, Figure 2, Figure 3, Figure 4, Figure 5 and Figure 6 and Table 1). In this study, the implementation of machine learning algorithms in data analyses brings the correct identification of one or more inherent features of each one of the samples involved in this study; this is observed from dendrograms, PCA, and confusion matrix calculations. Then, the predicted class in which a new sample (bacteria species FTIR spectra) is classified in the spatial region is filled in the PCA for a window interval due to the specific features of the sample. They are detected and classified by the statistical methodology implemented in the machine learning algorithms.

Then, they are analyzed in the PCA to give us the visualization of this classification by the features associated with the chemical interval group and the presence/absence of antibiotic susceptibility in the sample studied. The last is performed thanks to the accuracy of the data obtained from the FTIR spectrum and high accuracy in the determination and verification of the parameters in supervised/unsupervised learning methods. The features cited before are associated with the fingerprint signatures in the FTIR spectra of the bacteria species in the analyzed interval windows of Carbohydrate (900–1200 cm^−1^), Fatty Acids (2800–3100 cm^−1^), and Protein (1500–1800 cm^−1^) [11,12,14,16,17,19,20,24,25,26,29,46,89,90,91,92,93].

### 4.6. Machine Learning Algorithms

To find and determine the effect of the antibiotics with machine learning algorithms, we start with the FTIR absorption spectra of *S. aureus* that have been acquired following the procedure report by Soares et al. in [24,25] with resistance-induced strains protocol by Soares et al. in [26]. Data analyses were developed following the steps of the protocol of Naumann et. al. [46]: the calculation of the second derivative for each spectrum individually; normalization by the maximum value of FTIR absorption intensity; the extraction of window interval group. The FTIR spectrum obtained after implementing this procedure to Carbohydrate, Fatty Acid, and Protein groups are shown in Figure 1 and Figures S1–S3 in Supplementary Materials. Each group was analyzed separately to determine its specific contribution to the antibiotic resistance profile. All these data processing was developed by our owner code developed in MATLAB (R2021b) [32].

Following the methodology developed in the previous study [20], we introduced the data obtained previously to the supervised/unsupervised machine learning algorithms applied to spectrum analysis. This allowed us to extend the application of our model to a broader range of bacterial species. Then, the original dataset obtained from FTIR spectra was run through multivariate statistical analyses [94] to supervised/unsupervised machine learning algorithms] [20,49,79,95,96,97]. In addition, deep learning [10,30,33,77,78,79,98] was implemented in this study for the identification of antibiotic-resistant microorganisms.

The antibiotic resistance identification developed from the data from the Gram-positive and Gram-negative species studied here was run through evaluation in the confusion matrix. In them, the diagonal entries give the numbers of correct predictions for each species in the Carbohydrate, Fatty Acid, and Protein groups, respectively. The data obtained from the FTIR spectrum are tested in a calculation loop many times to obtain a real and confident fraction of the true negatives that were correctly identified, and a fraction of the positives that truly are positive were classified and correctly identified. The last were evaluated until they obtained high parameter values of sensitivity, specificity, and precision in the calculations developed by confusion matrix methodology.

The statistical tools implemented and machine learning algorithms were crucial in analyzing and extracting information, starting with data cleaning to enhancing class definition [62,72,99]. There are many statistical tools to be implemented in the analysis of data; to choose them, it is necessary to have clear data meaning that the system to be studied has implicit data [62,68,72,99,100,101]

To mention, some of them have the following tools and procedures: classification, balanced sampling, statistical sample representation, histograms, regression, three diagrams, distance functions, distance metrics, generalization accuracy, accuracy metric, balanced accuracy, classification rules, typicality, coverage lemma, data mining, pattern mining, relational mining, and statistical behavior [62,72,77,99,102]. The process developed here implemented the multiple-criteria decision analysis golden rule introduced by Yager [103] to provide a scalar representative value to the data analyses [62,68,71,72,77,82,84].

Due to the essential multiclass classification of species in each window interval (Carbohydrate (900–1200 cm^−1^), Fatty Acids (2800–3100 cm^−1^), and Protein (1500–1800 cm^−1^)), we have implemented the classification statistical process for the features of the samples. It was carried out with the implementation of statistical and multivariate analysis methods in the supervised and unsupervised machine learning algorithms in all the data.

The study has implemented the data analyses and graph-based and the affinity propagation spectral clustering and dendrograms, and these were performed by mean of the implementation of machine learning supervised/unsupervised algorithms [11,19,26,29,34,36,41,57,59,60,61,63,67,68,74,81,85,87,93,98,100,101,104,105,106,107].

Affinity propagation spectral clustering was selected due to its ability to efficiently handle large datasets and identify representative examples. The hierarchical method lets obtain the data spectra to be introduced to the PCAs and confusion matrix algorithms. It lets us observe the special feature’s behavior of the dataset in the window of chemical groups in each bacteria studied, in correspondence with a high accuracy level with the Gram-positive and Gram-negative bacterium species studied here.

The statistical process is performed independently in each FTIR spectra of the bacterium species across the three intervals in the FTIR spectra. The data analyses and machine learning algorithms have been developed and built in the R Project for Statistical Computing (4.2.3) [33,34,35,36,37,38,39,40,41].

Our results obtained with the use of machine learning algorithms, joined to statistical methods in the analyses and distribution of the data coming from the FTIR spectra developed here, in the fingerprint regions showed an excellent way to obtain a classification of data with high accuracy, and a high efficacy in the algorithms developed for this task, and obtained marks of antibiotic classification and prediction in an accurate way to the microorganism studied.

## 5. Conclusions

We have originally carried out the seminal work demonstrating the capability of the method in *S. aureus* [4]. This way proves that the identification of antibiotic resistance by machine learning algorithms, developed previously in *S. aureus* [20], works with other microorganisms and has obtained the potential to be a broad technique.

From our statistical results, we identify specific features associated with patterns that differentiate microorganisms into the species studied. The results bring a safe alternative to the detection of antibiotic resistance in microorganisms.

FTIR spectral analysis combined with machine learning algorithms provides methodological tools for the rapid identification of antibiotic-resistant microorganisms. In addition, it is achieved with high accuracy, while cluster analysis helps reveal new information about microorganism features associated with resistance mechanisms diversity in bacteria.

PCA and confusion matrix applied to FTIR spectra can contribute to the identification of biochemical features that could help differentiate microorganisms. Thus, combining FTIR spectroscopy with machine learning in antibiotic resistance in microorganism study would provide information to enhance diagnostic and supply tools for antibiotic resistance research. Also, this could open new ways for scientific discovery in the identification and characterization of microorganisms.

The results obtained with machine learning algorithms applied in this study for four microorganisms indicate cluster analysis results based on specific spectral signatures. It implies that within the studied microorganisms, there are significant variations in the Carbohydrate, Fatty Acid, and Protein composition or the structure of bacterial cells.

The statistical results identify specific features that are associated with patterns that differentiate the microorganisms and species and identify their similarities to the groups in the database built in the previous study [20].

## Figures and Tables

**Table 1:** Identification of antibiotic resistance results from confusion matrix implementation on the FTIR absorption spectra database of *S. aureus*. Correlation between the machine learning analysis results across various FTIR spectral regions for biomolecules (Carbohydrates, Fatty Acids, and Proteins) and antibiotic susceptibility (AMO, GEN, and ERY), Control, and microorganism species.

Sample Group	Bacteria	Molecular Window Interval Groups	Antibiotic Resistance Susceptibility Identified	Result Machine Learning Identification	Real Antibiotic Susceptibility
Gram-positive	*Streptococcus pyogenes*	Carbohydrates	AMO	AMO	Accurate
		Fatty acids	AMO		
		Proteins	AMO		
	*Streptococcus mutans*	Carbohydrates	GEN	ERY	Accurate
		Fatty acids	ERY		
		Proteins	ERY		
Gram-negative	*Escherichia coli*	Carbohydrates	GEN	GEN, ERY, and AMO	Accurate
		Fatty acids	ERY		
		Proteins	AMO		
	*Klebsiella pneumoniae*	Carbohydrates	CONTROL	ERY	Accurate
		Fatty acids	ERY		
		Proteins	ERY		

## Data Availability

The data presented in this study are available upon request from the corresponding authors.

## Supplementary Materials

The following supporting information can be downloaded at: https://www.mdpi.com/article/10.3390/antibiotics13090821/s1. Supplementary Materials can be found in the entire sample FTIR spectra of the bacterium sample for the four species studied here, dendrograms, PCAs, and calculation results of the confusion matrix in the antibiotic resistance identification process in the chemistry interval windows for the species Control, AMO, ERY, and GEN.
