# Supplementary material for: Machine Learning in FTIR Spectrum for the Identification of Antibiotic Resistance: A Demonstration with Different Species of Microorganisms
Source: Antibiotics (Basel). 2024 Aug 30;13(9):821. doi: 10.3390/antibiotics13090821 (PMC11428736; doi:10.3390/antibiotics13090821)
Supplement: Supplementary file 1 [file antibiotics-13-00821-s001.zip › antibiotics-3098027-supplementary.pdf]

Supplementary of the Paper (Article)

Machine Learning in FTIR Spectrum for the Identification of Antibiotic Resistance: A Demonstration with Different Species of Microorganisms

Claudia Patricia Barrera Patiño [1,*], Jennifer Machado Soares [1], Kate Cristina Blanco [1] and Vanderlei Salvador Bagnato [1,2,*]

[1] São Carlos Institute of Physics, University of São Paulo, Avenida Trabalhador São-Carlense nº 400, Parque Arnold Schimidt, Sao Carlos CEP 13566-590, SP, Brazil; jennifer.soares@usp.br (J.M.S.); kateblanco@ifsc.usp.br (K.C.B.)

[2] Biomedical Engineering, Texas A&M University, 400 Bizzell St., College Station, TX 77843, USA

* Correspondence: cpbarrerap@ifsc.usp.br (C.P.B.-P.); vander@ifsc.usp.br (V.S.B.); bagnatovs@tamu.edu

The result of evaluating the FTIR spectrum of bacteria species implemented in this study on groups associated with carbohydrates, fatty acids, and proteins, is shown into the next figures in complement to the results shown into the main article main body. This material is in concordance and complement with the results shown into the main body article paper. Here, we shown the results obtained with tested and evaluated supervised/unsupervised machine learning algorithms implemented into the study for the species group: Control, AMO, GEN, ERY, and Gram-positive and Gram-negative bacterium species, to determine structural variations of FTIR spectra into chemical interval windows groups. This methodologic shown the results in the identification of antibiotic resistance.

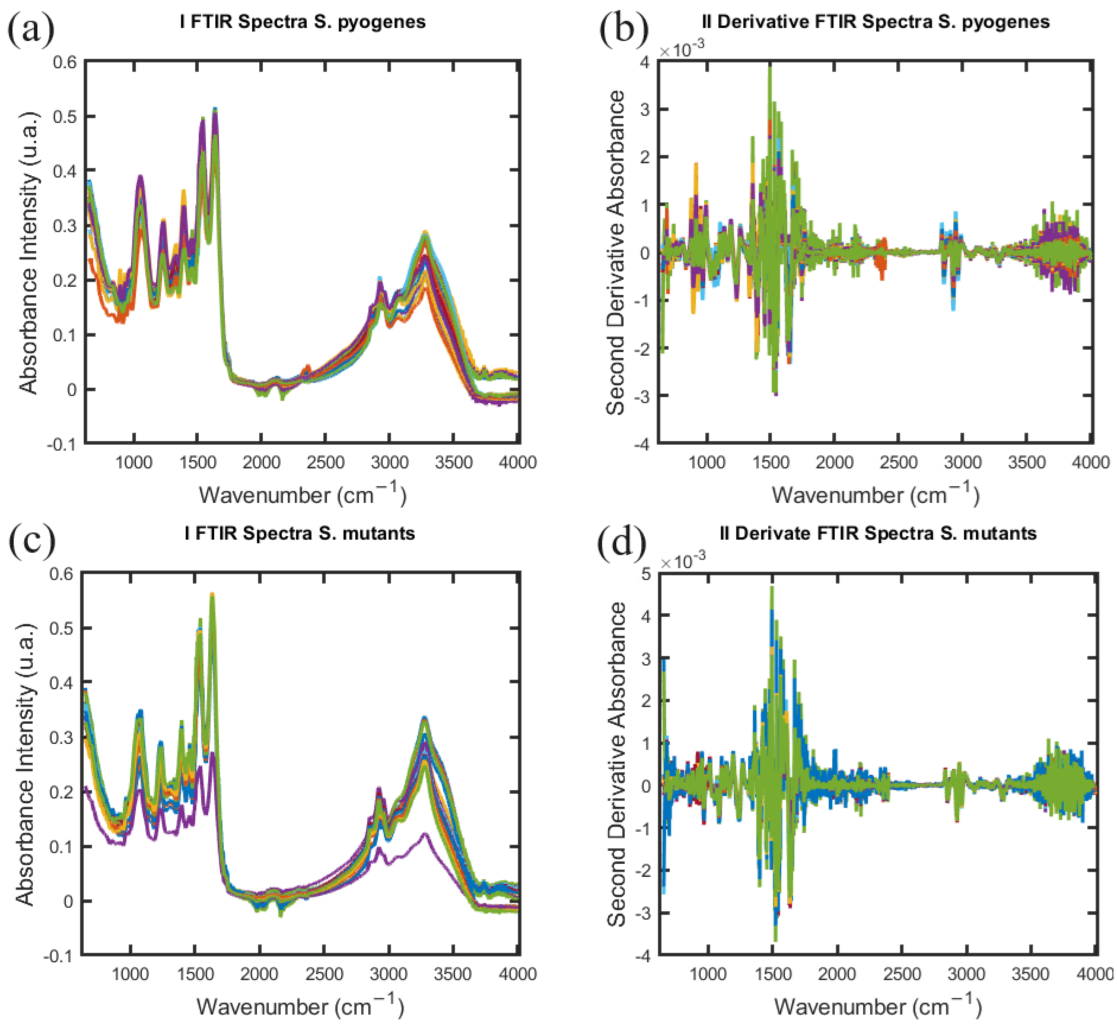

Figure S1. FTIR spectra to *S. pyogenesis* (a-b) and *S. mutans* (c-d), with the process to second derivative absorbance.

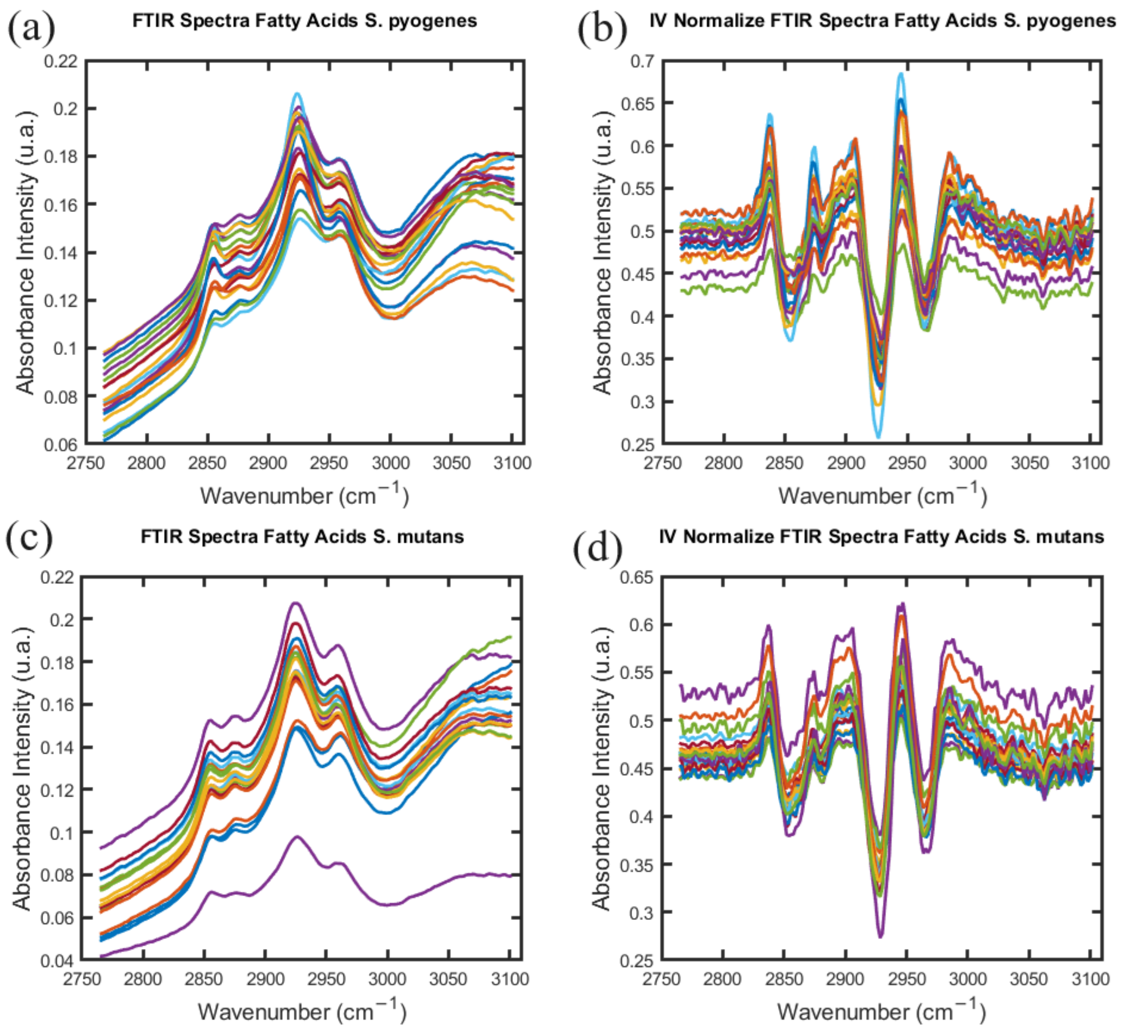

Figure S2. FTIR spectra to fatty acids interval windows to *S. pyogenesis* (a-b) and *S. mutans* (c-d), with the normalize absorbance obtained to this interval region to each bacteria specie.

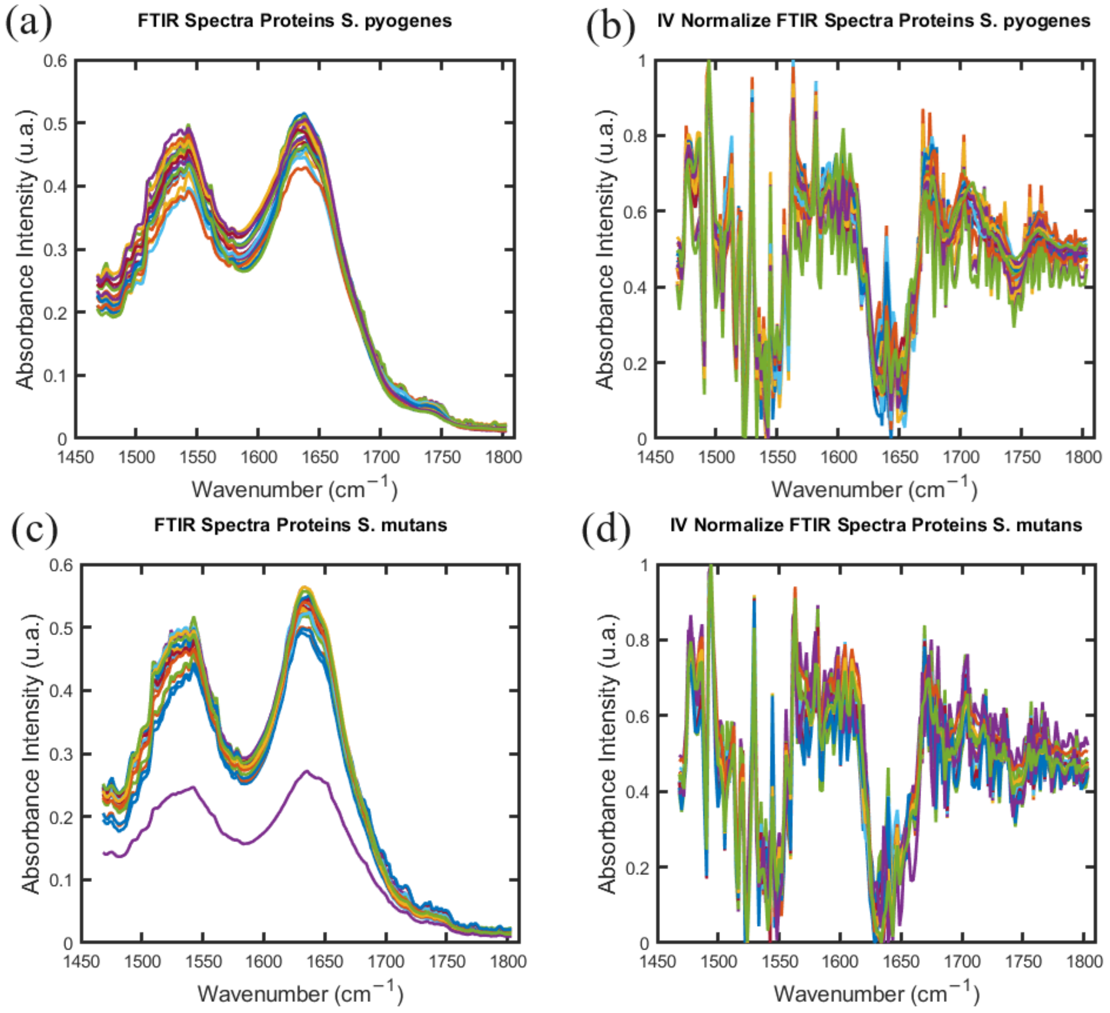

Figure S3. FTIR spectra to protein interval windows to *S. pyogenesis* (a-b) and *S. mutans* (c-d), with the normalize absorbance obtained to this interval region to each bacteria specie.

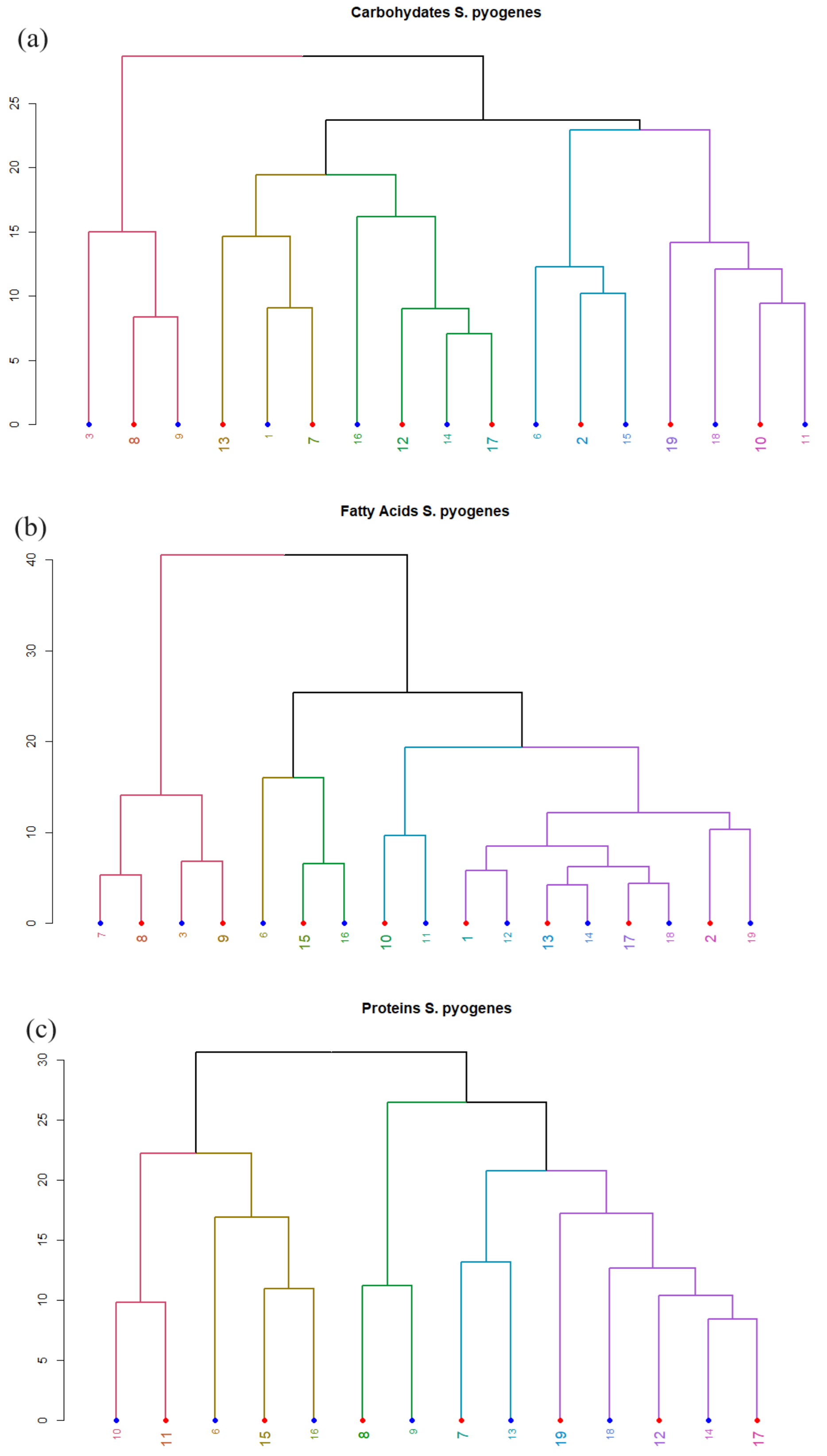

Figure S4. Dendrogram analyses to *S. pyogenes* bacteria samples for twenty FTIR spectra into the carbohydrate, fatty acids, and protein intervals windows.

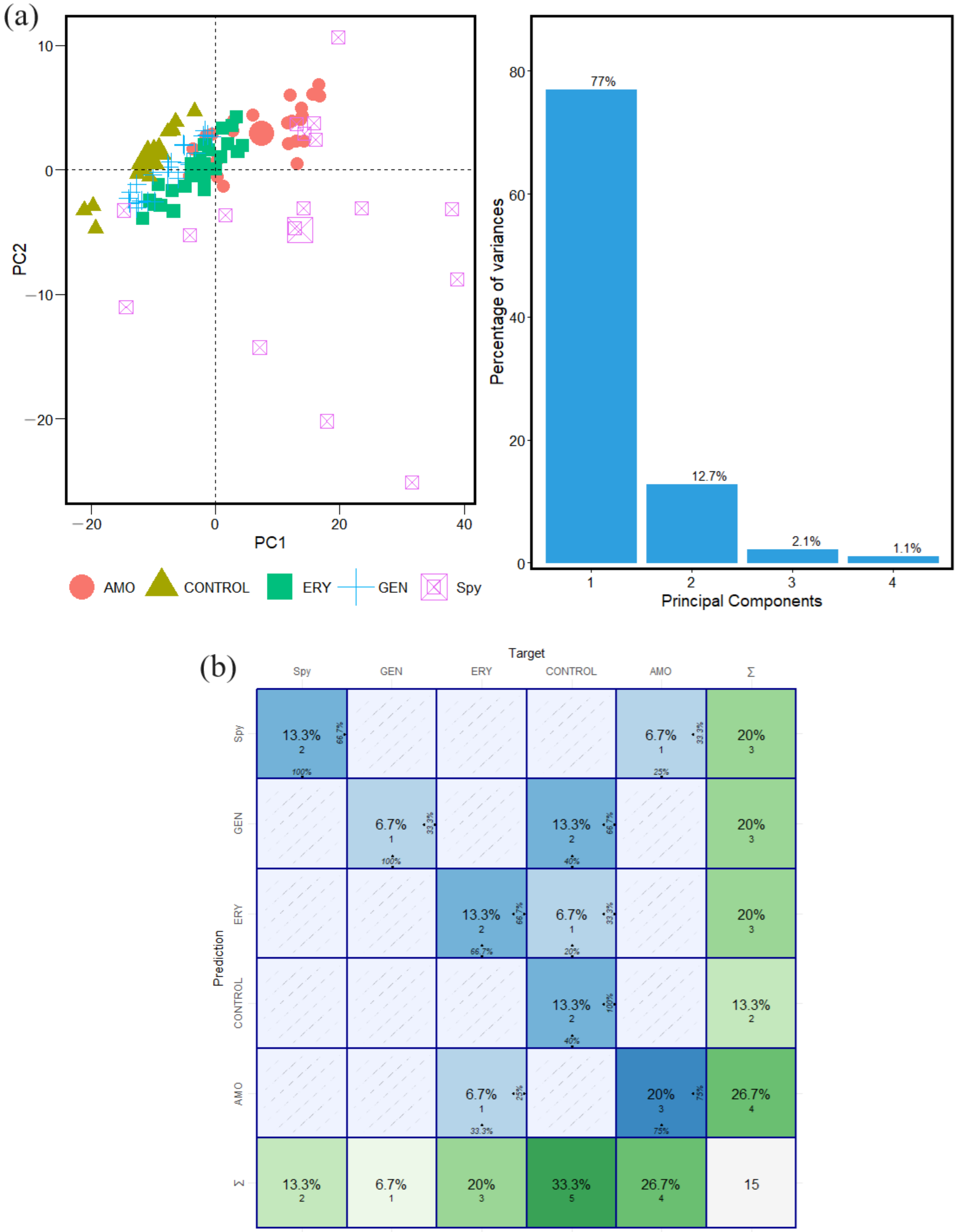

Figure S5. Classification steps and machine learning analyses for twenty FTIR spectra of *S. pyogenes* bacteria samples into fatty acids windows interval. (a) PCA calculation and statistical variance results. (b) Confusion matrix calculation results.

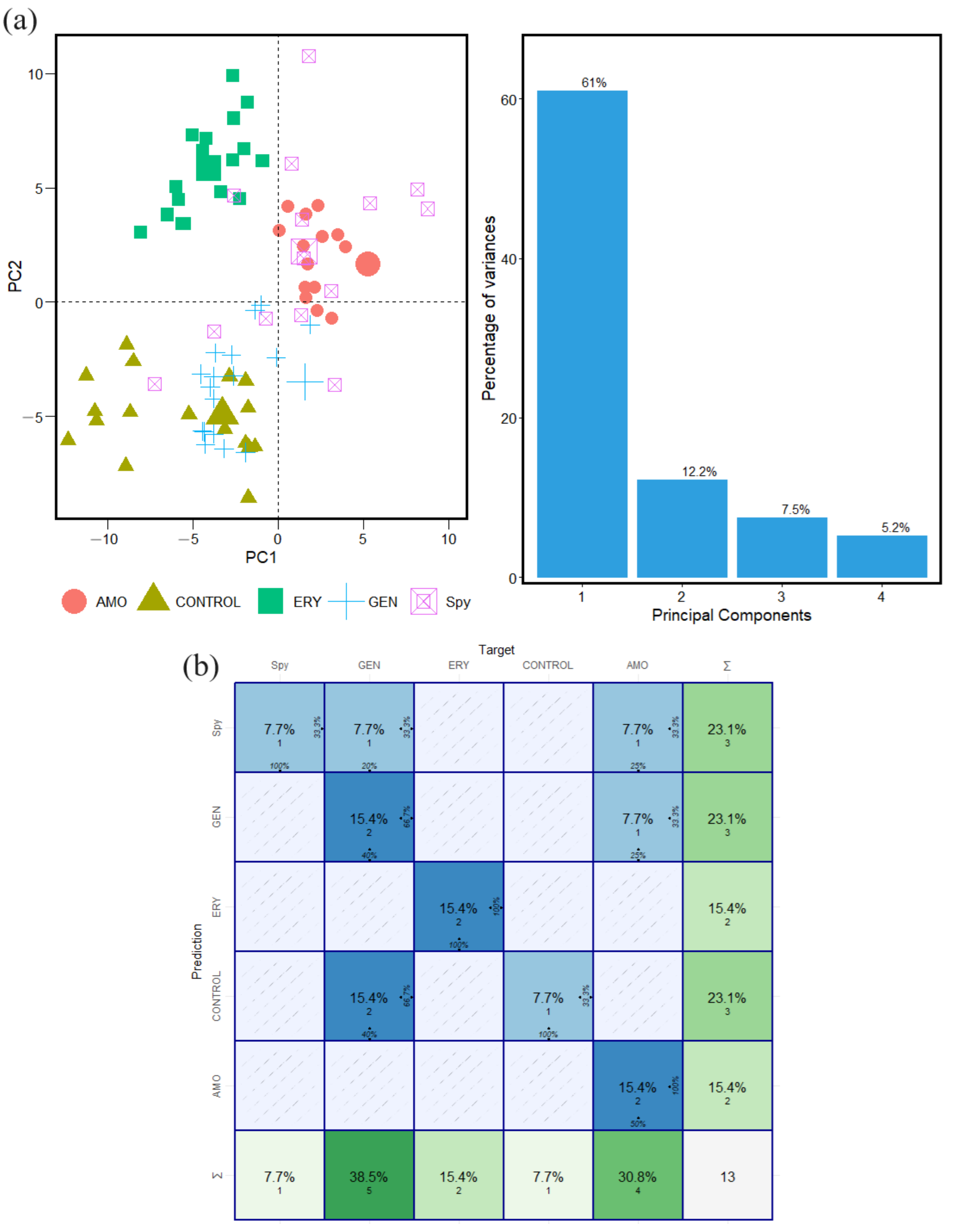

Figure S6. Classification steps and machine learning analyses for twenty FTIR spectra of *S. pyogenes* bacteria samples into protein windows interval. (a) PCA calculation and statistical variance results. (b) Confusion matrix calculation results.

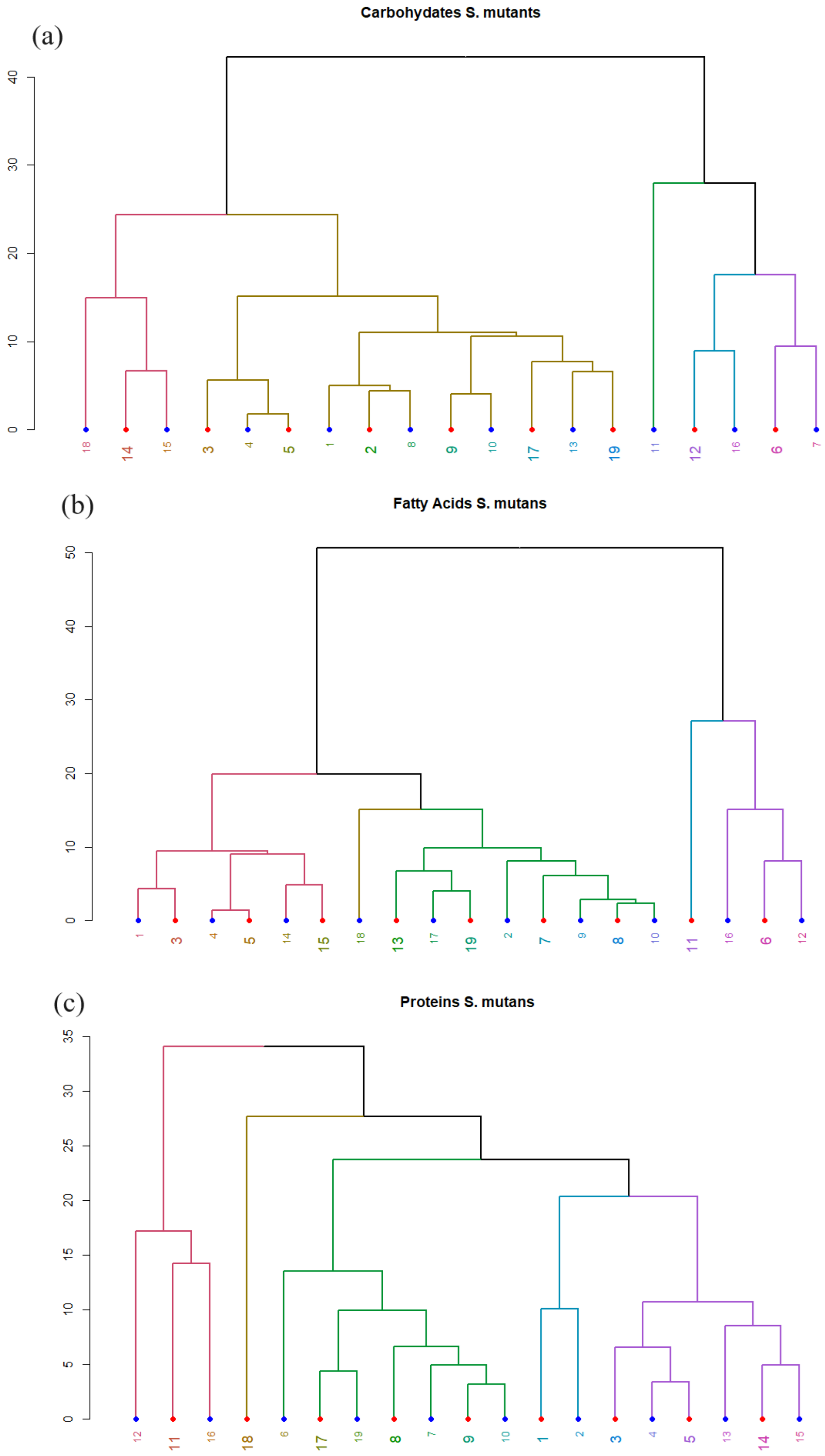

Figure S7. Dendrogram analyses to *S. mutans* bacteria samples for twenty FTIR spectra into the carbohydrate, fatty acids, and protein intervals windows.

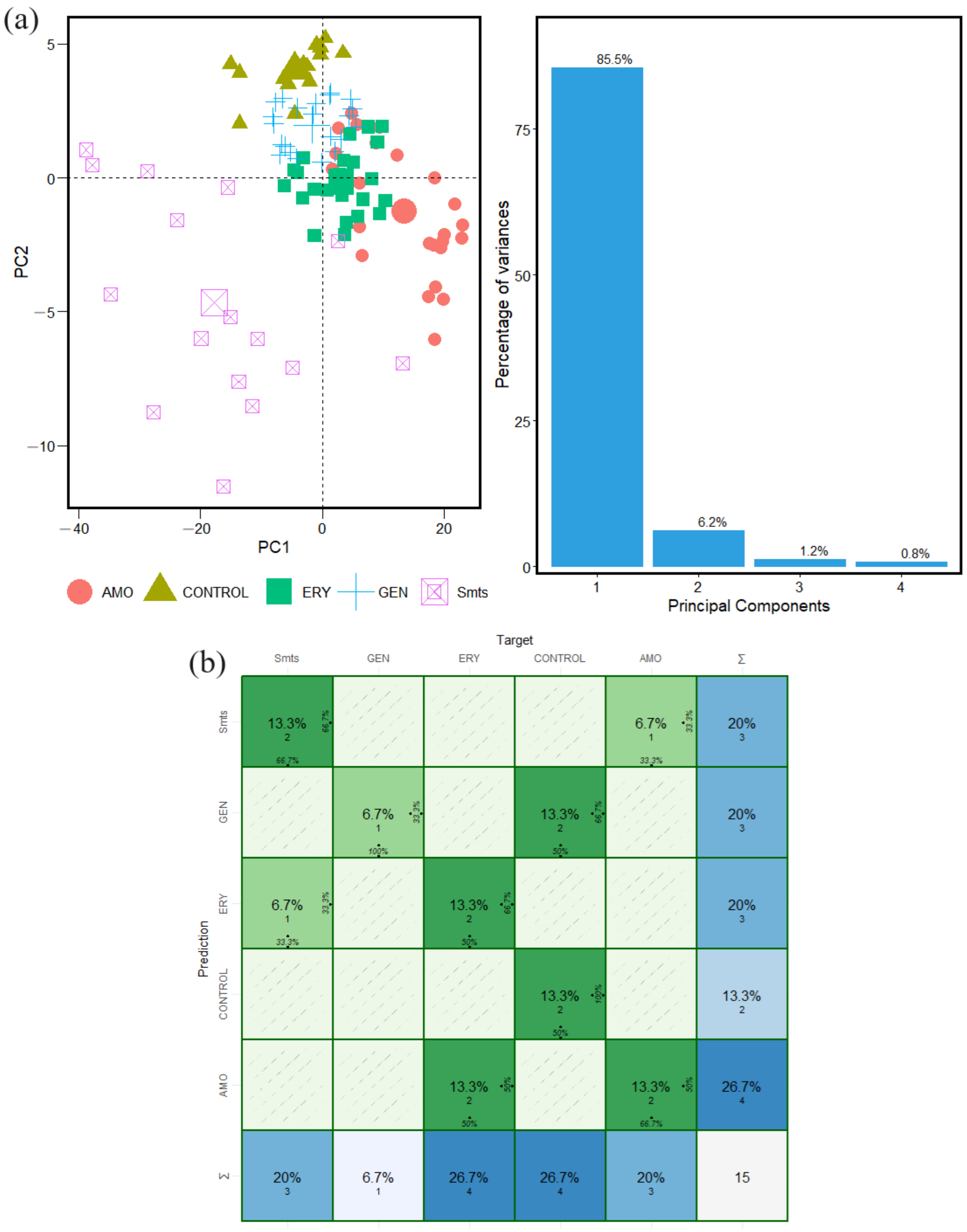

Figure S8. Classification steps and machine learning analyses for twenty FTIR spectra of *S. mutans* bacteria samples into fatty acids windows interval. (a) PCA calculation and statistical variance results. (b) Confusion matrix calculation results.

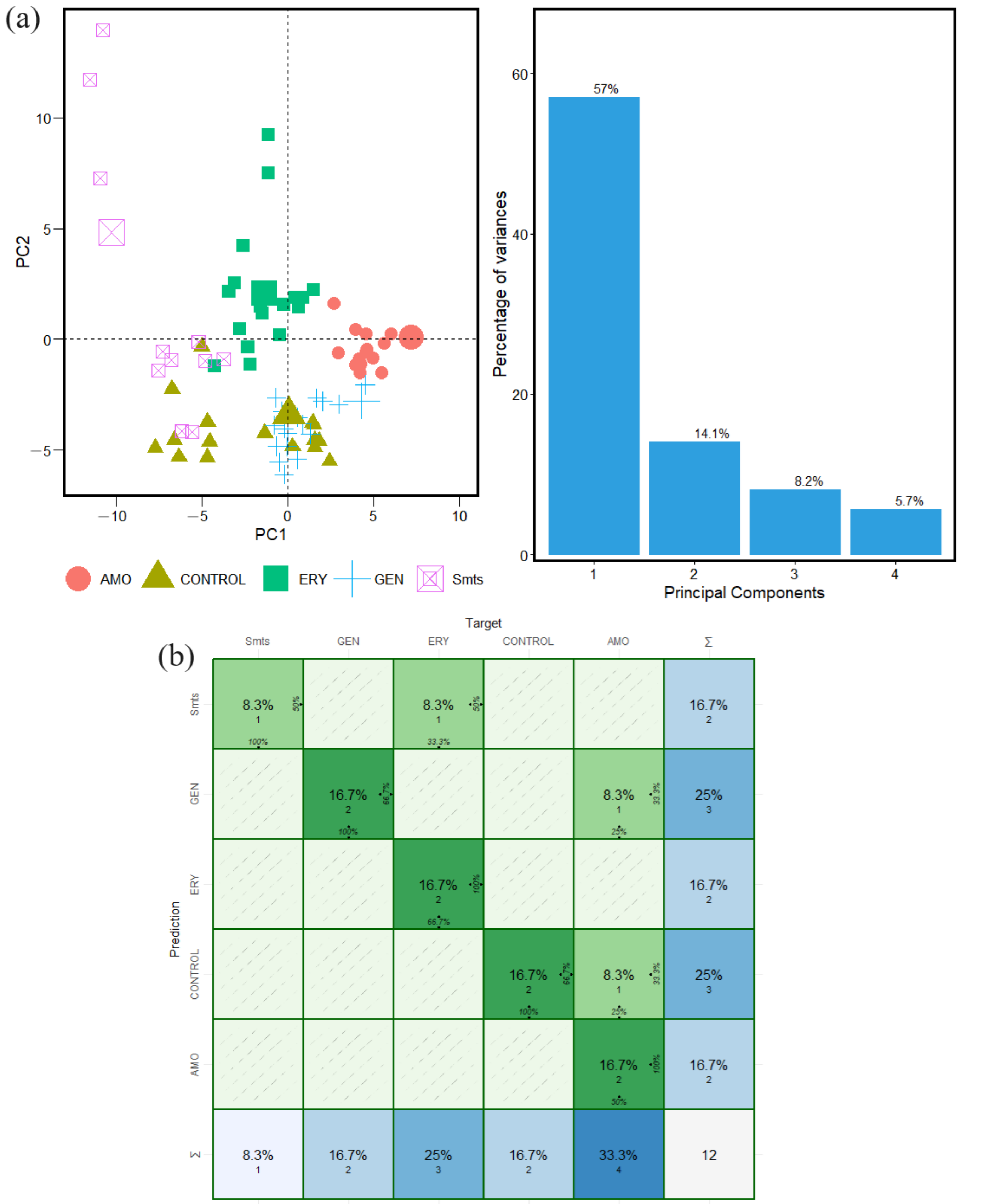

Figure S9. Classification steps and machine learning analyses for twenty FTIR spectra of *S. mutans* bacteria samples into protein windows interval. (a) PCA calculation and statistical variance results. (b) Confusion matrix calculation results.

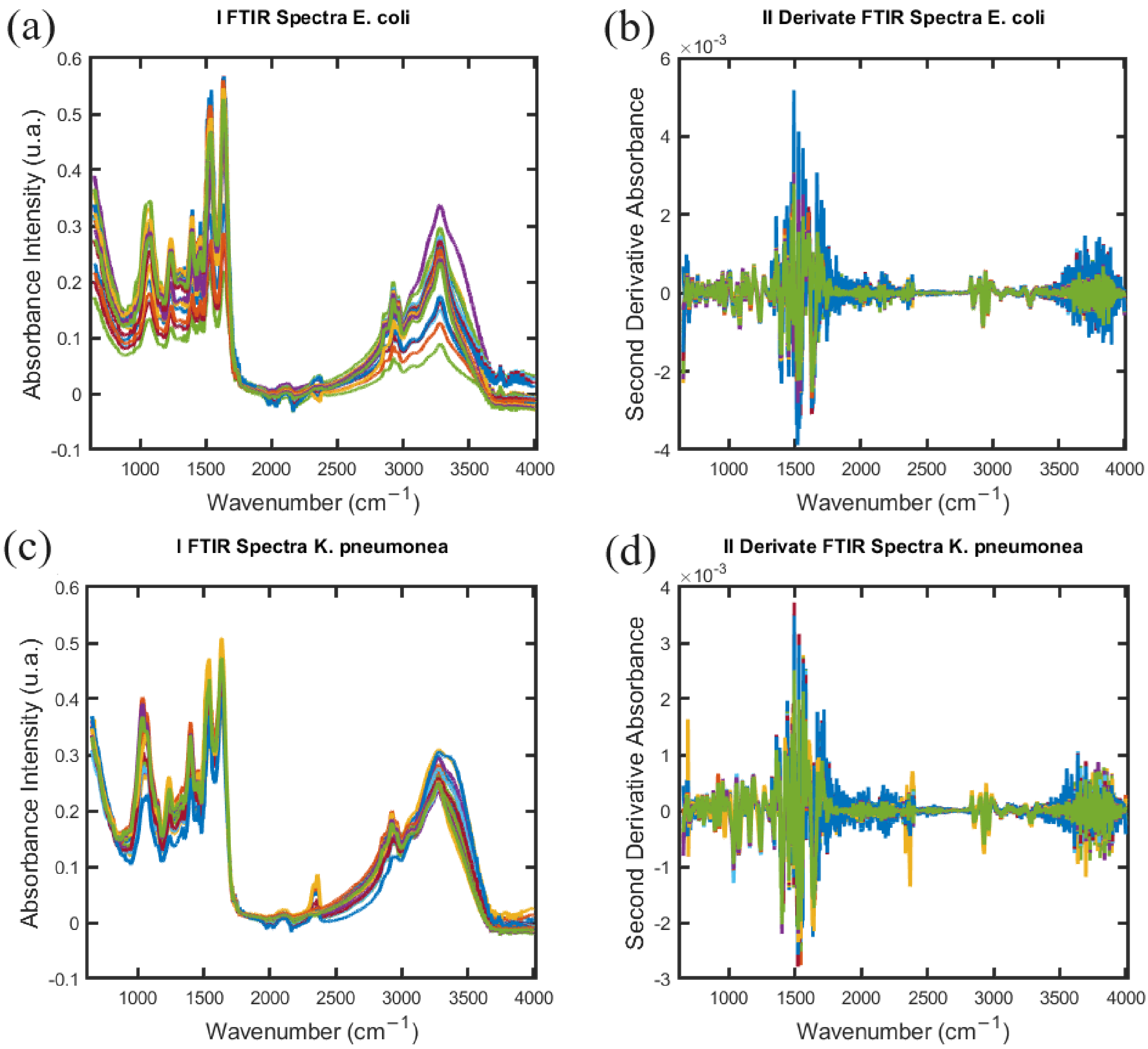

Figure S10. FTIR spectra to *E. coli* (a-b) and *K. pneumoniae* (c-d), with the process to second derivative absorbance.

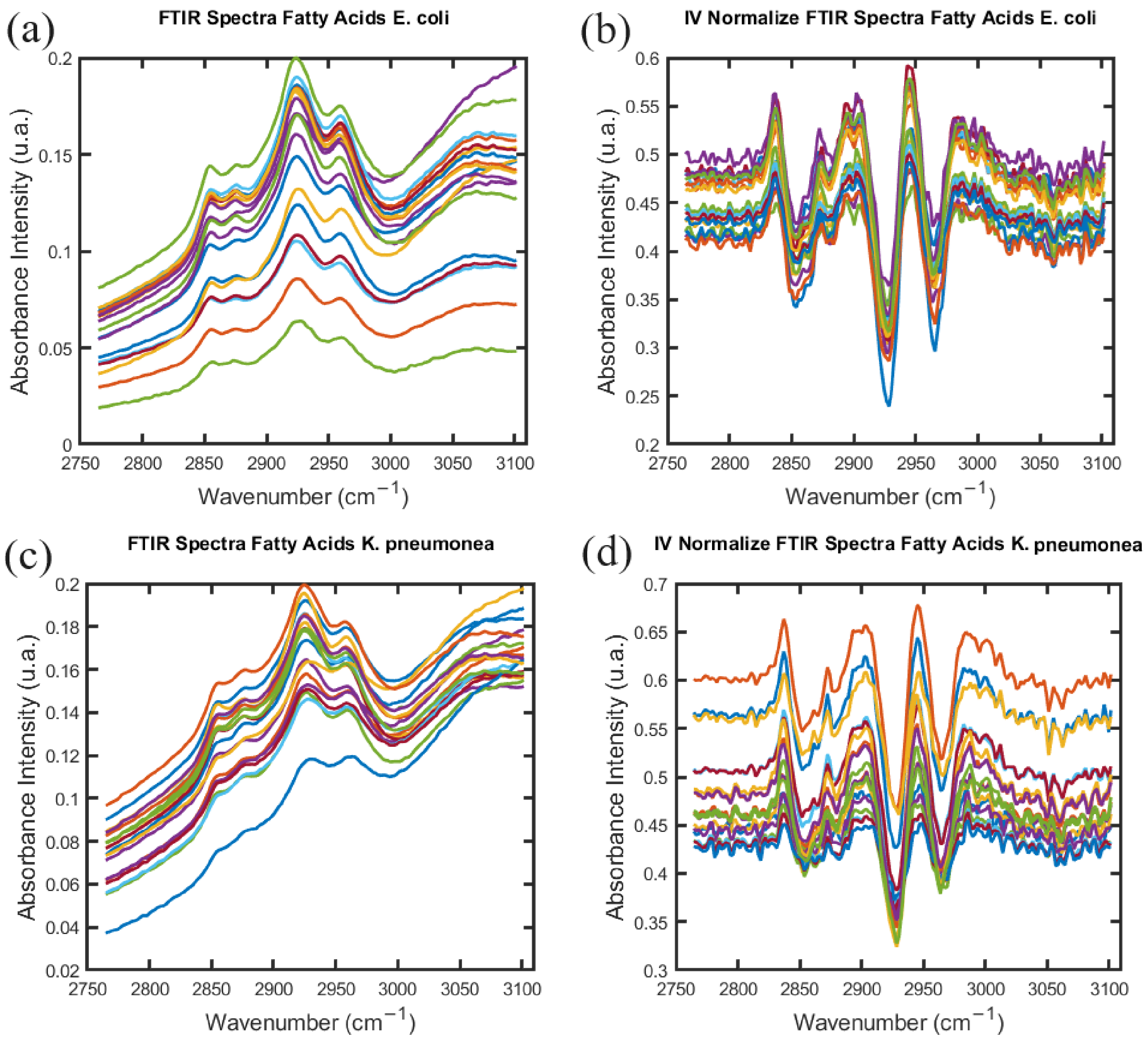

Figure S11. FTIR spectra to fatty acids interval windows to *E. coli* (a-b) and *K. pneumoniae* (c-d), with the normalize absorbance obtained to this interval region to each bacteria specie.

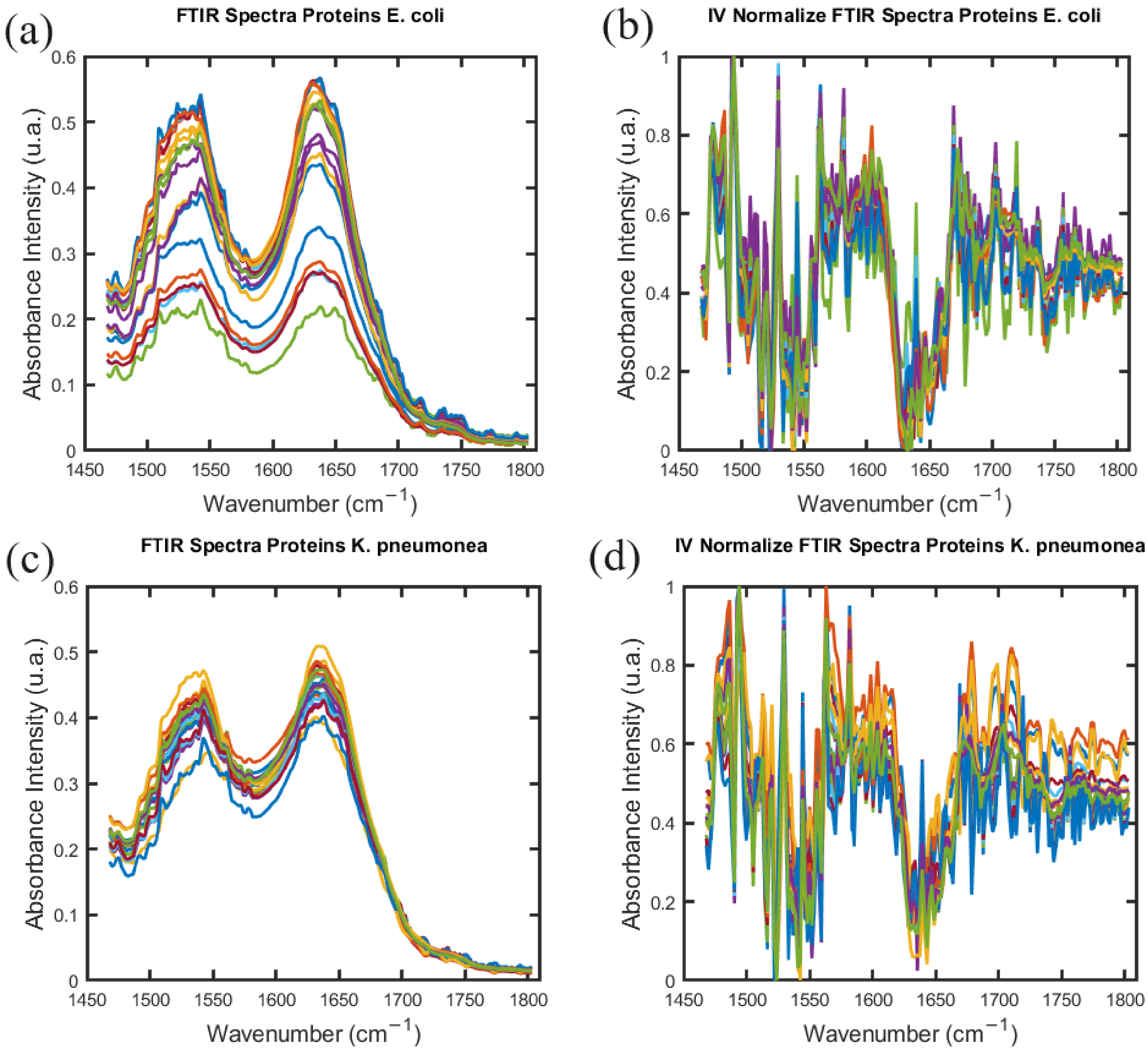

Figure S12. FTIR spectra to protein interval windows to *E. coli* (a-b) and *K. pneumoniae* (c-d), with the normalize absorbance obtained to this interval region to each bacteria specie.

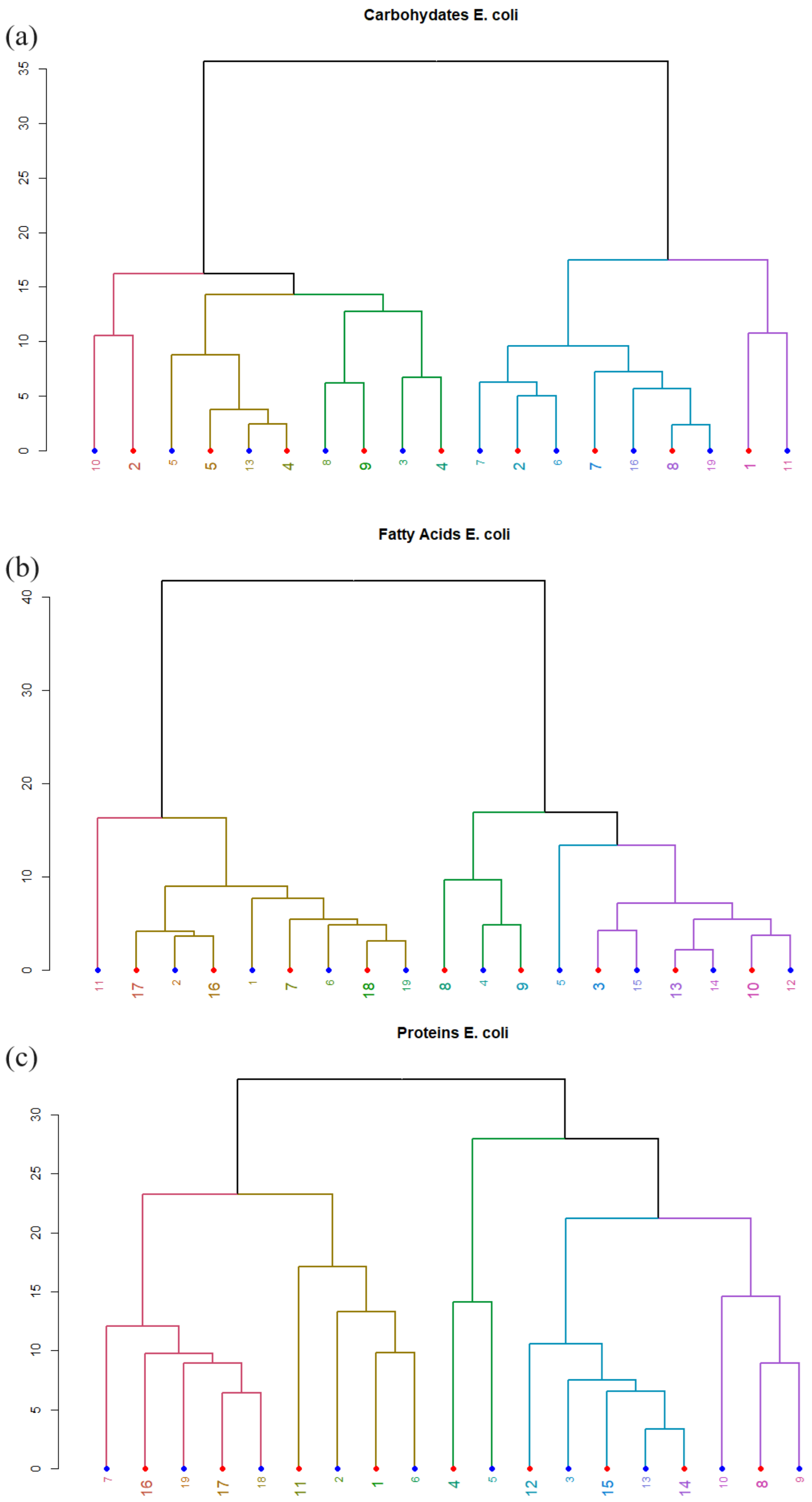

Figure S13. Dendrogram analyses to *E. coli* bacteria samples for twenty FTIR spectra into the carbohydrate, fatty acids, and protein intervals windows.

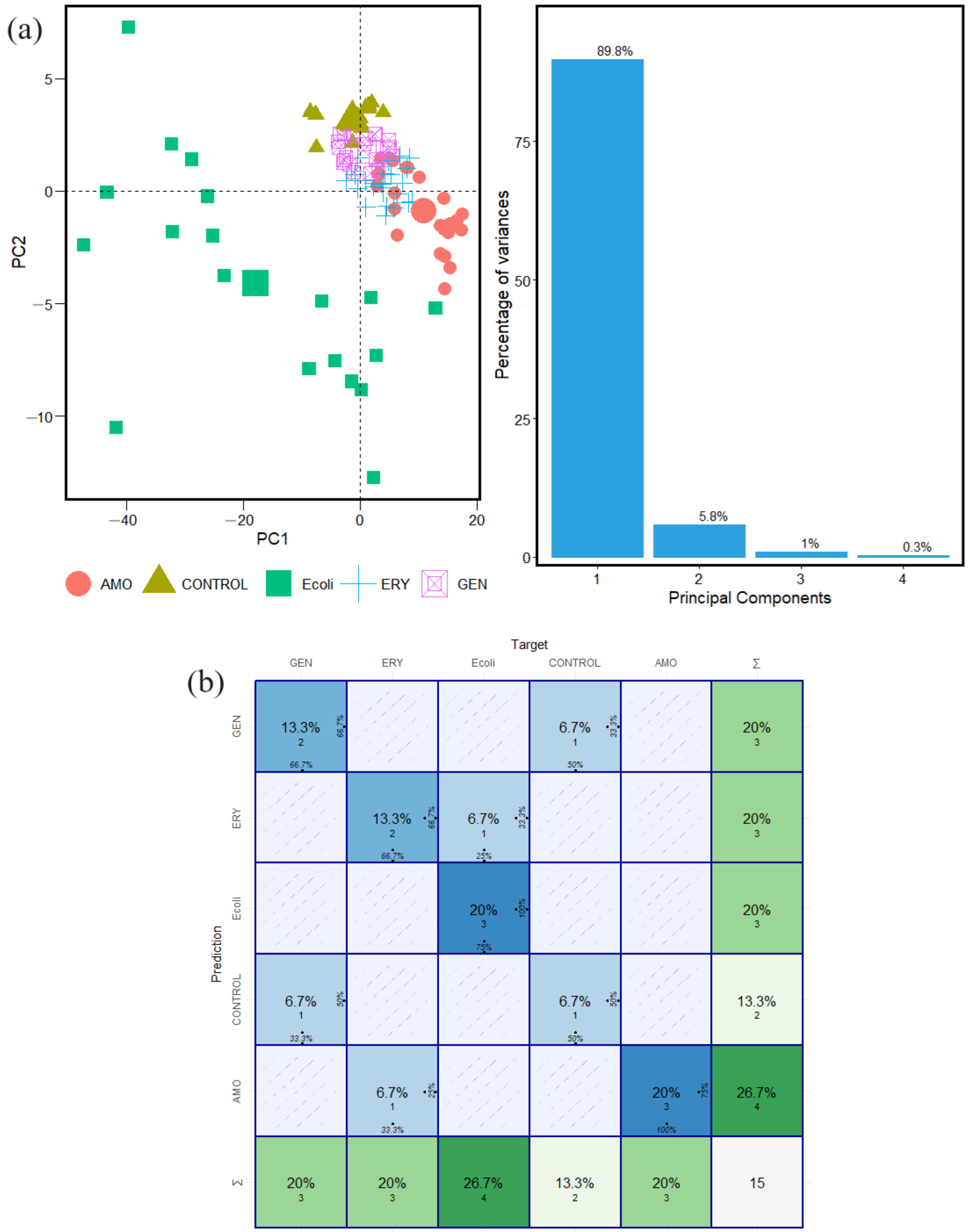

Figure S14. Classification steps and machine learning analyses for twenty FTIR spectra of *E. coli* bacteria samples into fatty acids windows interval. (a) PCA calculation and statistical variance results. (b) Confusion matrix calculation results.

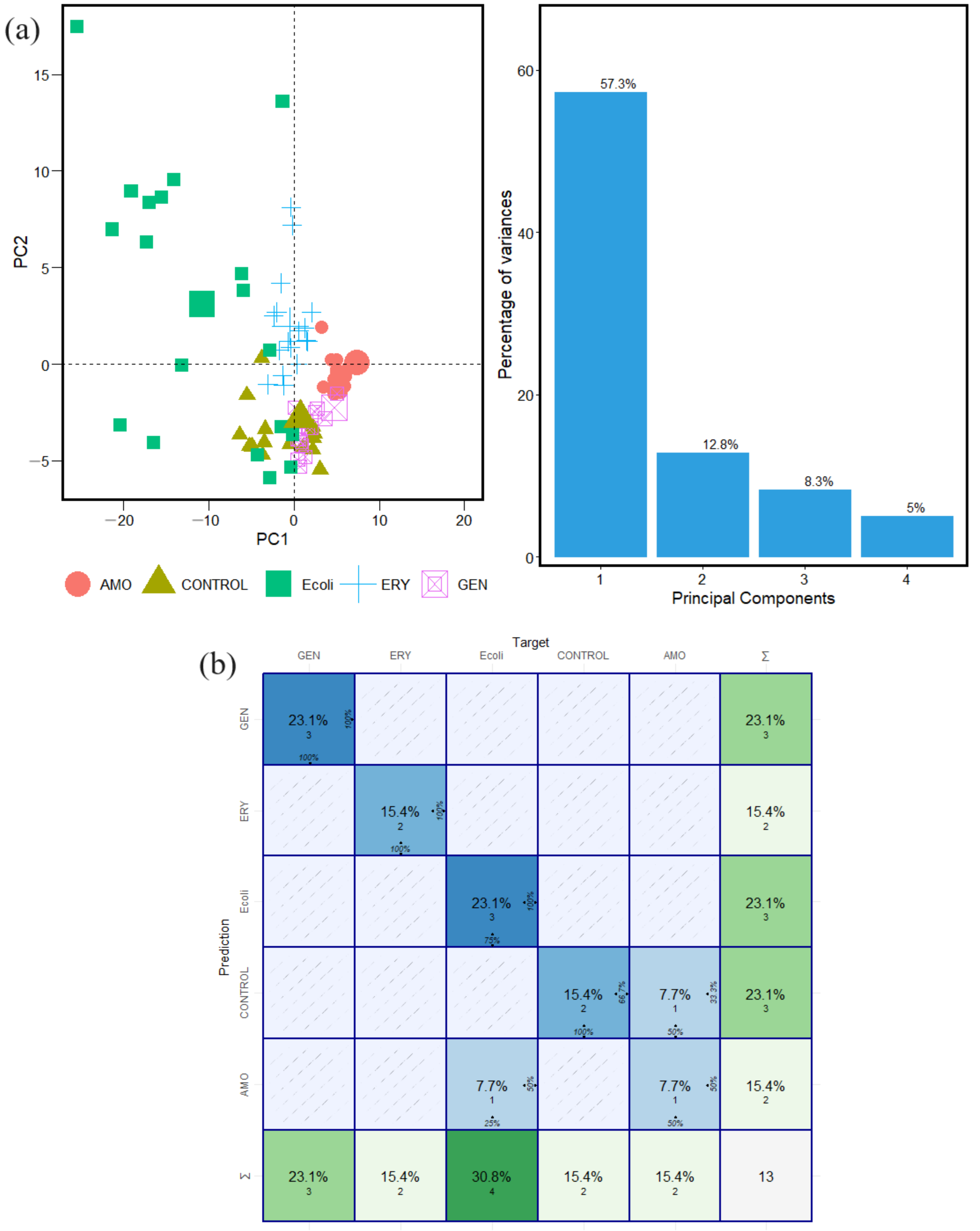

Figure S15. Classification steps and machine learning analyses for twenty FTIR spectra of *E. coli* bacteria samples into protein windows interval. (a) PCA calculation and statistical variance results. (b) Confusion matrix calculation results.

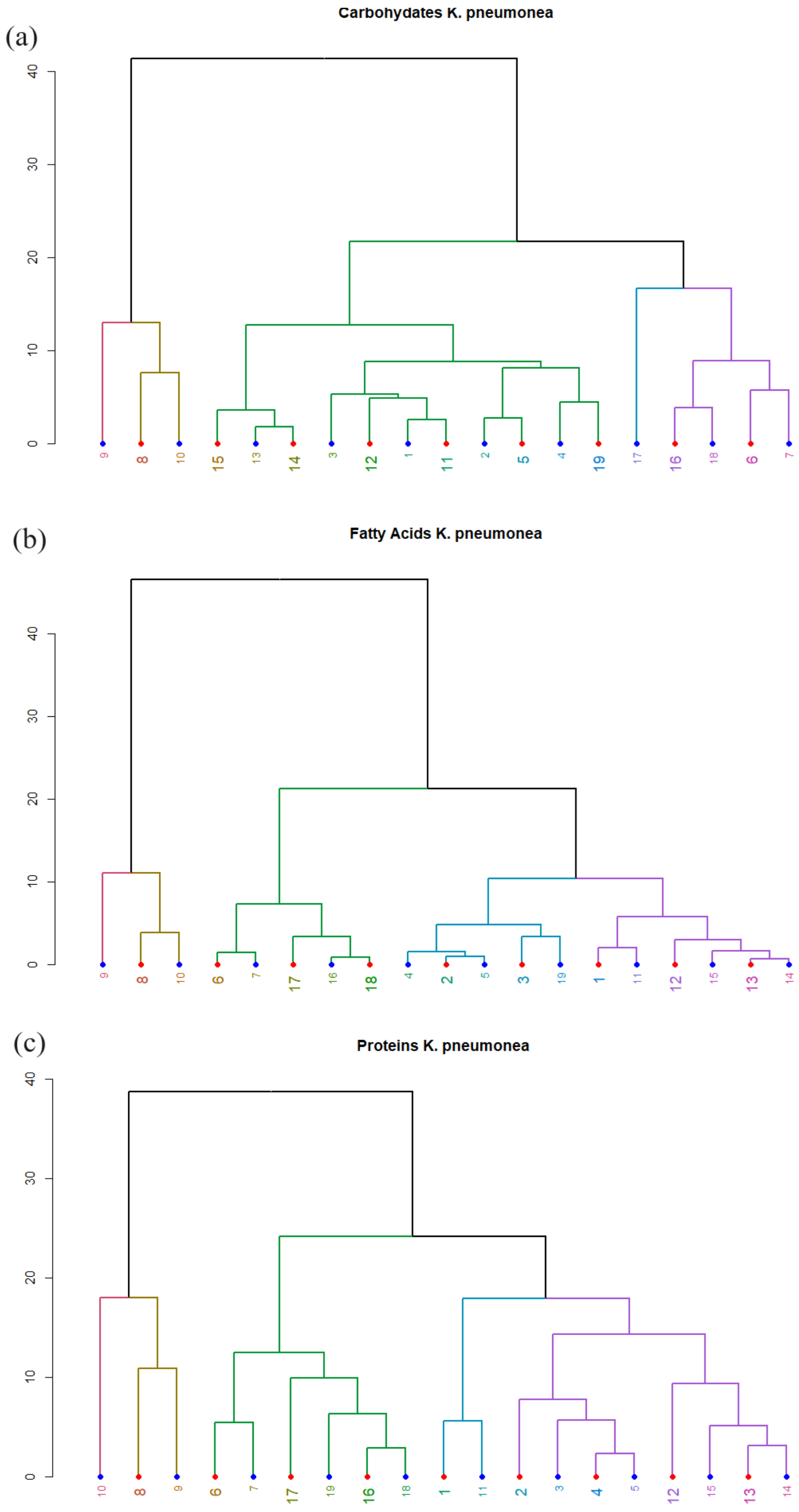

Figure S16. Dendrogram analyses to *K. pneumoniae* bacteria samples for twenty FTIR spectra into the carbohydrate, fatty acids, and protein intervals windows.

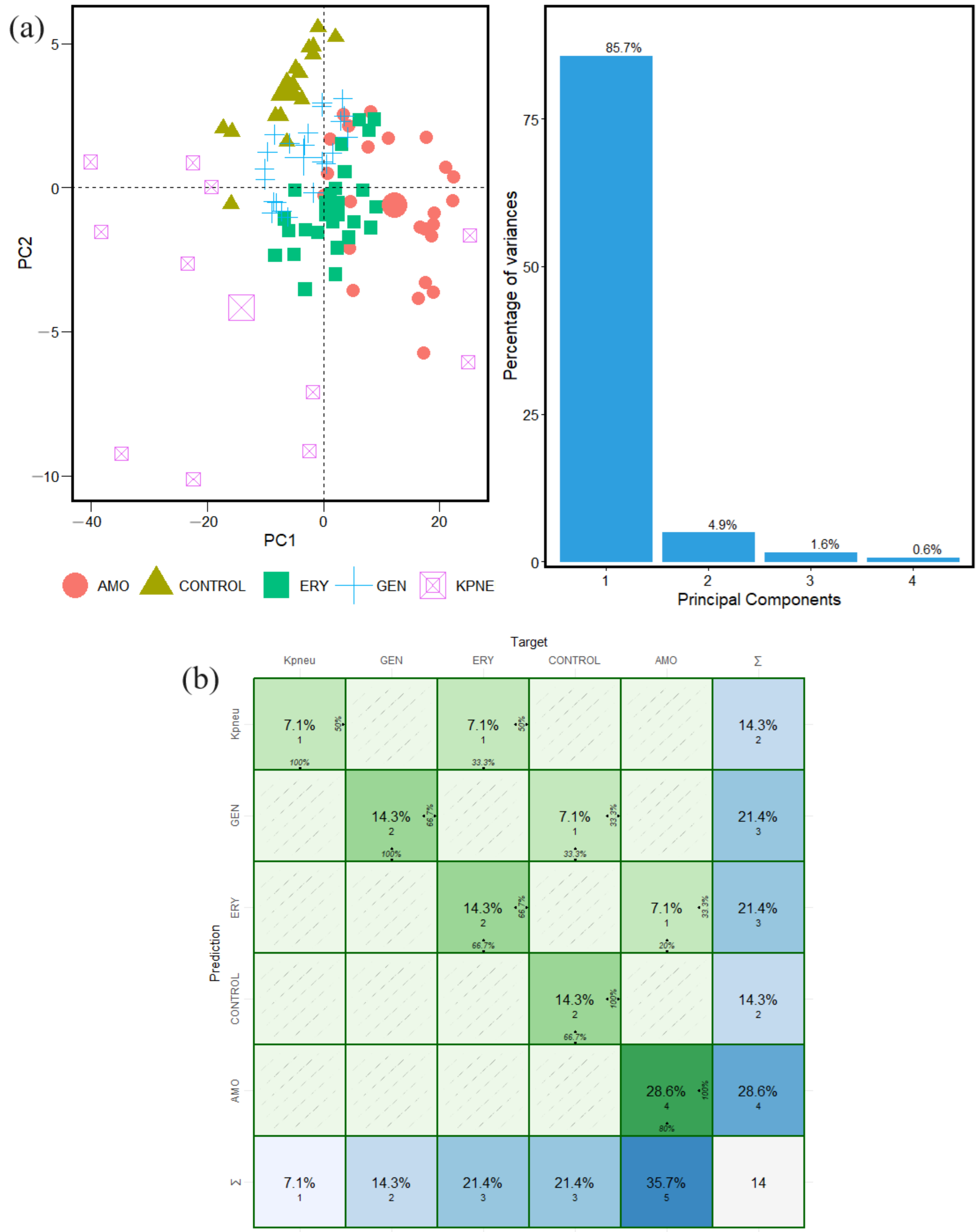

Figure S17. Classification steps and machine learning analyses for twenty FTIR spectra of *K. pneumoniae* bacteria samples into fatty acids windows interval. (a) PCA calculation and statistical variance results. (b) Confusion matrix calculation results.

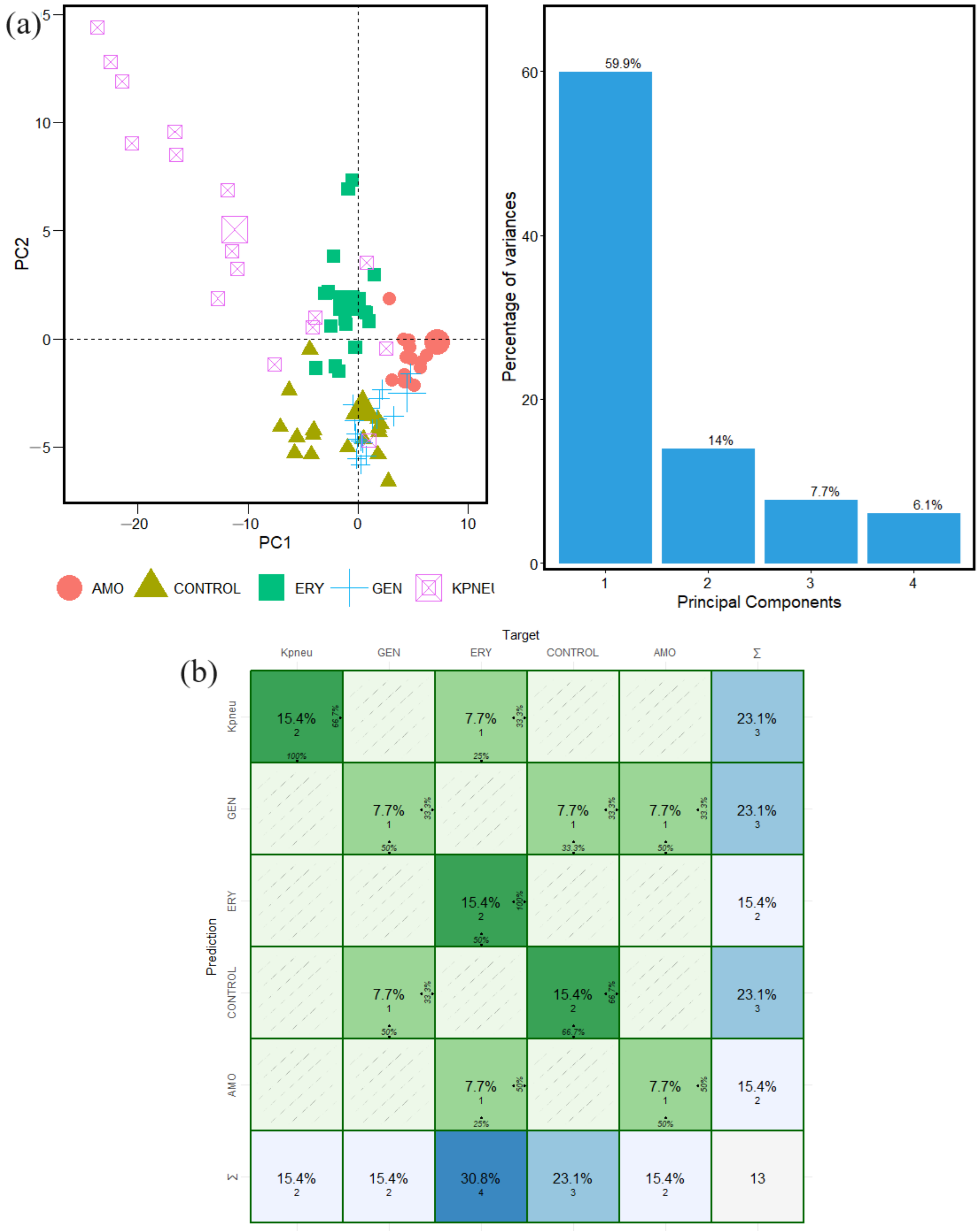

Figure S18. Classification steps and machine learning analyses for twenty FTIR spectra of *K. pneumoniae* bacteria samples into protein windows interval. (a) PCA calculation and statistical variance results. (b) Confusion matrix calculation results.
